# Distinct Arp2/3 isocomplexes drive axonal actin ring assembly and integrity

**DOI:** 10.1126/sciadv.aec9522

**Published:** 2026-09-25

**Authors:** Ana Rita Costa, Teresa Lopes, Luís P. Rodrigues, João M. Rocha, José C. Mateus, Maria Leonor Moura, Christoph Spahn, Marko Lampe, Naoko Kogata, António J. Pereira, Paulo Aguiar, Pedro Brites, Paula Sampaio, Michael Way, Monica M. Sousa

**Affiliations:** ^1^ i3S-Instituto de Investigação e Inovação em Saúde, Universidade do Porto, Porto, Portugal.; ^2^ Rudolf Virchow Center-Center for Integrative and Translational Bioimaging, Julius-Maximilians-University Würzburg, Würzburg, Germany.; ^3^ Advanced Light Microscopy Facility, European Molecular Biology Laboratory, Heidelberg, Germany.; ^4^ Light Microscopy Core Facility, German Cancer Research Center (DKFZ), Heidelberg, Germany.; ^5^ The Francis Crick Institute, London, UK.; ^6^ CF-UM-UP-Centre of Physics of the Universities of Minho and Porto, Universidade do Minho, Braga, Portugal.; ^7^ Department of Infectious Disease, Imperial College, London, UK.

## Abstract

The actin-related protein 2/3 (Arp2/3) complex is a central nucleator of branched actin filaments, essential for numerous cellular processes. Using superresolution imaging, we demonstrate that Arp2/3 is a component of the membrane periodic skeleton (MPS)—a highly ordered actin ring-spectrin lattice in the submembrane axonal cytoskeleton. While generally viewed as a single entity, functional specialization of the Arp2/3 complex arises from isoform diversity of three of its seven subunits. Moreover, isoform-specific perturbations revealed that distinct Arp2/3 isocomplexes orchestrate successive stages of MPS actin ring development. While Arp2/3 isocomplexes containing ARPC1A and ARPC5 are essential for actin ring assembly during early MPS formation, those with ARPC1B and ARPC5L are key to maintaining actin rings in mature axons. These findings demonstrate that beyond linear actin, branched actin filaments contribute to the MPS nanoarchitecture and uncover a switch in Arp2/3 isocomplex composition that is required to ensure actin ring assembly and stability.

## INTRODUCTION

Actin plays a fundamental role in shaping cell morphology and driving dynamic cellular processes. In neurons, the organization and remodeling of the actin cytoskeleton are crucial for early events such as polarization, differentiation, and the growth of axons and dendrites (*1*, *2*). Superresolution imaging of neurons led to the discovery of a previously unrecognized actin cytoskeleton structure—the axonal membrane periodic skeleton (MPS) (*3*). The MPS consists of submembrane actin rings interconnected by bipolar spectrin tetramers, resulting in a regular ring periodicity of ∼190 nm (*3*). This actin-spectrin architecture assembles shortly after axon specification, propagating from proximal regions, including the axon initial segment (AIS), to distal regions of unmyelinated and myelinated axons (*4*). Although the MPS is also found in dendrites, including the necks of dendritic spines (*5*), its presence in this neuronal compartment varies in extent (*4*, *6*). To date, the MPS has been identified in all neuronal types examined, across both central and peripheral nervous systems, spanning species from *Caenorhabditis elegans* and *Drosophila* to rodents and humans, underscoring its evolutionary conservation and significance for neuronal function (*3*, *4*, *7*–*9*). The MPS contributes to multiple aspects of neuronal biology, such as providing structural support to axons (*10*), restricting endocytosis along proximal axons (*11*), organizing voltage-gated channels within the axonal membrane (*4*), regulating axon diameter by binding α-adducin and nonmuscle myosin II (*8*, *9*, *12*), stabilizing axonal microtubules (*13*), and serving as a transient signaling platform (*14*) [reviewed in (*15*)]. The importance of this unique actin-spectrin organization is further emphasized by the fact that its disassembly precedes axon degeneration (*16*, *17*). The early model for actin organization within the MPS proposed that actin rings are composed of short, capped actin filaments on the basis of the detection of the actin-capping protein α-adducin within the structure (*3*). More recently, correlative superresolution and electron microscopy of unroofed proximal axons revealed actin structures composed of two long, intertwined, unbranched filaments, which may represent segments of individual actin rings (*18*). Nevertheless, the molecular details underlying axonal actin ring nucleation and assembly remain largely elusive.

To further elucidate the molecular composition and functions of the MPS, coimmunoprecipitation and mass spectrometry were recently used to identify new components of the MPS (*19*). This analysis identified subunits of the actin-related protein 2/3 (Arp2/3) complex, as well as cortactin, a stabilizer of Arp2/3-mediated actin branches (*19*, *20*). The Arp2/3 complex is a highly conserved, seven-subunit protein complex that plays a central role in regulating the actin cytoskeleton organization in multiple biological contexts (*21*–*23*). When the Arp2/3 complex is activated by nucleation-promoting factors (*24*, *25*), it binds to the side of an existing (mother) actin filament and nucleates a new (daughter) filament, forming a characteristic branched structure, with elongation occurring at the barbed end of the daughter filament (*26*, *27*). The Arp2/3 complex is composed of two actin-related proteins—Arp2 and Arp3—and five additional subunits, ARPC1 through ARPC5, that ensure structural stability and function [reviewed in (*21*)]. Together, the coordinated actions of all seven subunits enable the Arp2/3 complex to nucleate branched actin filament networks, which are essential for key cellular processes such as migration, endocytosis, and immune synapse formation. Notably, the subunits Arp3, ARPC1, and ARPC5 exist as two different isoforms encoded by separate genes—Arp3 and Arp3B, ARPC1A and ARPC1B, and ARPC5 and ARPC5L, respectively, resulting in eight distinct Arp2/3 isocomplexes (*28*–*32*). The distinct cellular and physiological roles of Arp2/3 isocomplexes have been highlighted in recent studies, supporting the notion that these isoforms are not functionally interchangeable. For instance, loss of ARPC1B results in severe inflammation and immunodeficiency even if ARPC1A expression is increased (*33*). Likewise, while loss of ARPC5 causes multiple congenital anomalies and systemic inflammation (*33*–*36*), ARPC5L-deficient mice have no immune or inflammatory abnormalities (*36*), further supporting the notion that ARPC5- and ARPC5L-containing Arp2/3 isocomplexes are not exchangeable. Despite these findings, the relevance of distinct Arp2/3 isocomplexes in biology is in general overlooked as the Arp2/3 complex is largely regarded as a single entity.

Here, we examined the axonal MPS to investigate how Arp2/3 isocomplexes contribute to the organization of actin networks. Our results uncover a previously unrecognized role of distinct Arp2/3 isocomplexes in regulating the maturation of MPS actin rings. Furthermore, our findings support the notion that branched actin filaments are integral components of the MPS architecture.

## RESULTS

### Arp2/3 is a component of MPS actin rings

**Fig. 1.**
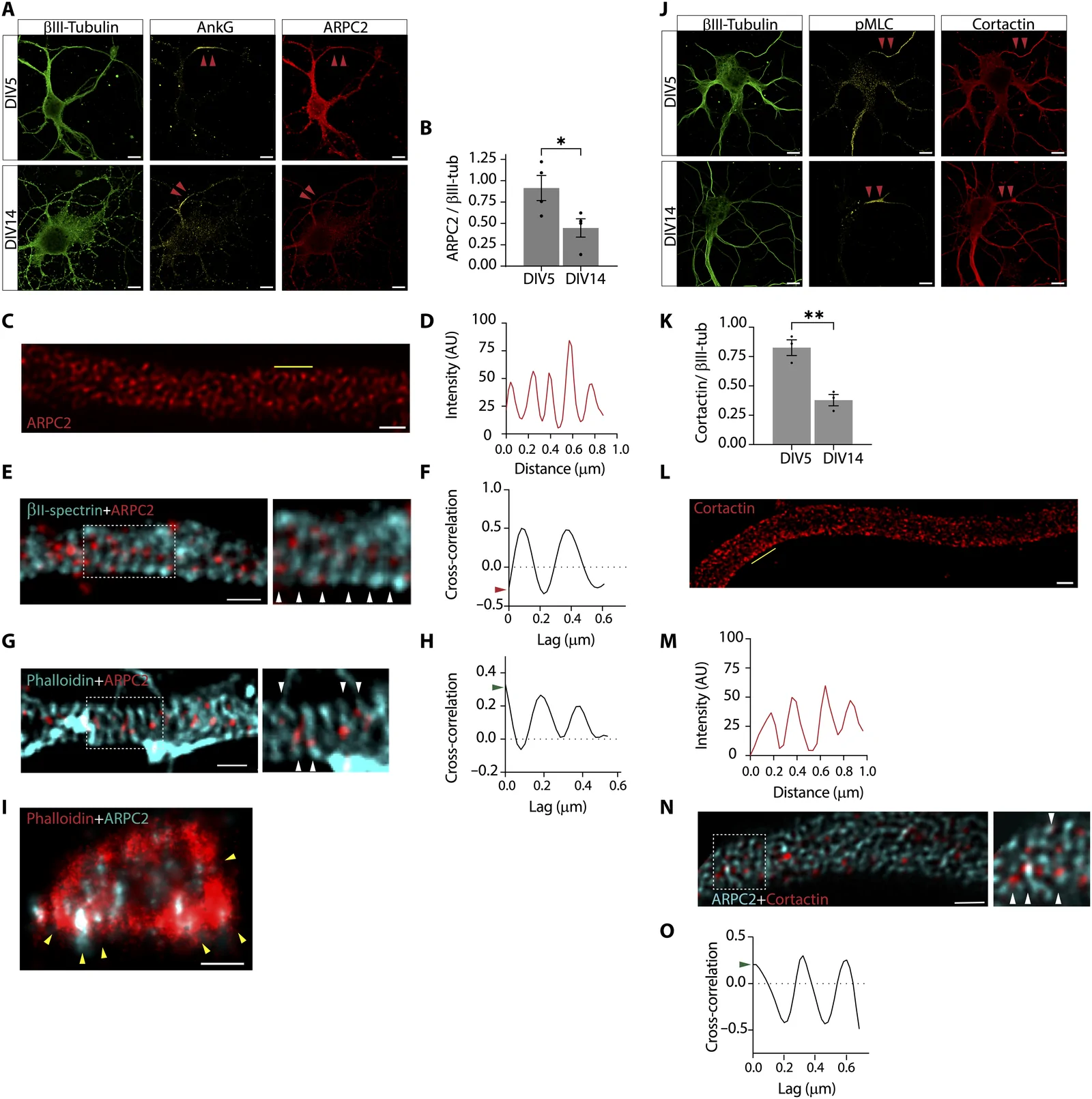
Axonal ARPC2 and cortactin have periodic distribution. (**A**) DIV5 and DIV14 hippocampal neurons stained for βIII-tubulin, AnkG (AIS marker), and ARPC2. Arrowheads highlight the axon. Scale bars, 10 μm. (**B**) Quantification of axonal ARPC2 intensity; *n* = 3 independent experiments (20 neurons per experiment). (**C**) Tau-STED of DIV5 hippocampal axon stained for ARPC2. Scale bars, 0.5 μm. (**D**) Fluorescence intensity profile across the yellow line in (C). AU, arbitrary units. (**E**) Tau-STED of DIV5 hippocampal axon stained for βII-spectrin (cyan) and ARPC2 (red). A zoom-in of the boxed region illustrates their periodic and alternating distribution (arrowheads). Scale bar, 0.5 μm. (**F**) Cross-correlation of fluorescence intensity profiles from the boxed region in (E). The arrowhead indicates a negative value at zero lag. (**G**) Tau-STED of hippocampal neurons stained with phalloidin (cyan) and ARPC2 (red). A zoom-in of the boxed region illustrates their periodic distribution (arrowheads). Scale bar, 0.5 μm. (**H**) Cross-correlation of fluorescence intensity profiles from the boxed region in (G). The arrowhead indicates a positive value at zero lag. (**I**) Correlative dSTORM/DNA-PAINT of hippocampal neurons stained for phalloidin (red) and ARPC2 (cyan). Arrowheads highlight the localization of ARPC2 within the actin ring. Scale bar, 0.2 μm. (**J**) DIV5 and DIV14 hippocampal neurons stained for βIII-tubulin, 2-phospho-MLC (pMLC; AIS marker), and cortactin. Arrowheads indicate the axon. Scale bars, 10 μm. (**K**) Quantification of axonal cortactin intensity; *n* = 3 independent experiments (15 neurons per experiment). (**L**) Tau-STED of DIV5 hippocampal axon stained for cortactin. Scale bars, 0.5 μm. (**M**) Fluorescence intensity profile across the yellow line in (L). (**N**) Tau-STED of DIV5 hippocampal axon stained for ARPC2 (cyan) and cortactin (red). A zoom-in of the boxed region illustrates periodic and superimposed distribution (arrowheads). Scale bar, 0.5 μm. (**O**) Cross-correlation of fluorescence intensity profiles from the boxed region in (N). The arrowhead indicates a positive value at zero lag. Results are the means ± SEM; unpaired *t* test; **P ≤ 0.01 and *P ≤ 0.05; ns, nonsignificant.

Previous analysis of proteins coimmunoprecipitated with the MPS components α-adducin, βII-spectrin, and αII-spectrin in cultured embryonic hippocampal neurons and adult mouse brains revealed multiple Arp2/3 complex subunits as possible MPS components (*19*). These findings prompted us to examine whether the Arp2/3 complex is a structural component of the MPS and to assess whether Arp2/3-dependent actin nucleation plays a critical role in MPS actin ring biology. We started by exploring the Arp2/3 subcellular distribution and nanoscale organization during hippocampal neuron development in culture. We analyzed neurons at 5 days in vitro (DIV5), as this represents the earliest time point at which the MPS can be clearly detected by stimulated emission depletion (STED) microscopy, and at DIV14, where the MPS is fully established along the entire axon. These two time points enable the comparison between early MPS formation and later maintenance of the periodic cytoskeleton. Ankyrin-G (AnkG) (*37*) or phospho–myosin light chain (pMLC) (*12*) were used to label the AIS and therefore define the axon. In rat hippocampal primary neurons, confocal imaging detected ARPC2, a core structural component of the complex, in all neuronal compartments at DIV5, with reduced intensity in mature axons at DIV14 (Fig. 1, A and B). Single-color STED imaging showed that ARPC2 exhibits a periodic distribution along the axon, with an average spacing of 182 ± 20 nm, consistent with the characteristic pattern of MPS components (Fig. 1, C and D). Two-color STED imaging revealed that ARPC2 alternates with βII-spectrin along the axon (Fig. 1E, white arrowheads, inset), as quantitatively supported by the negative cross-correlation at zero lag (Fig. 1F). Providing additional evidence that Arp2/3 is a component of the MPS, two-color STED imaging of ARPC2 and phalloidin showed that ARPC2 puncta colocalize with actin rings along the axon (Fig. 1G, white arrowheads, inset), consistent with positive cross-correlation analysis (Fig. 1H). To further resolve the spatial localization of ARPC2 relative to actin rings, correlative three-dimensional stochastic optical reconstruction microscopy (3D-STORM)/DNA point accumulation in nanoscale topography (DNA-PAINT) imaging was performed, revealing ARPC2 puncta within the rings (Fig. 1I, yellow arrowheads).

In addition to ARPC2, we investigated the nanoscale organization of cortactin, which bridges activated Arp2/3 to its nucleated “daughter” actin filament, making it a useful marker for branched actin (*20*). Similar to ARPC2, cortactin was detected in axons at DIV5, with reduced levels at DIV14 (Fig. 1, J and K). High-resolution single-color STED imaging showed that cortactin also has a periodic distribution along the axon, with an average spacing of 205 ± 11 nm (Fig. 1, L and M). Moreover, two-color STED imaging revealed that cortactin periodically colocalizes with ARPC2 along the axon (Fig. 1N, inset, white arrowheads). To quantify this spatial alignment, we performed cross-correlation analysis of their intensity profiles that showed a strong positive correlation at zero lag, indicating that cortactin and ARPC2 spatial fluctuations tend to colocalize along the axon (Fig. 1O). Together, the above findings demonstrate that the Arp2/3 complex and branched actin, confirmed by the presence of cortactin, are structural components of the MPS.

### Arp2/3 is critical for MPS actin ring assembly and maintenance

**Fig. 2.**
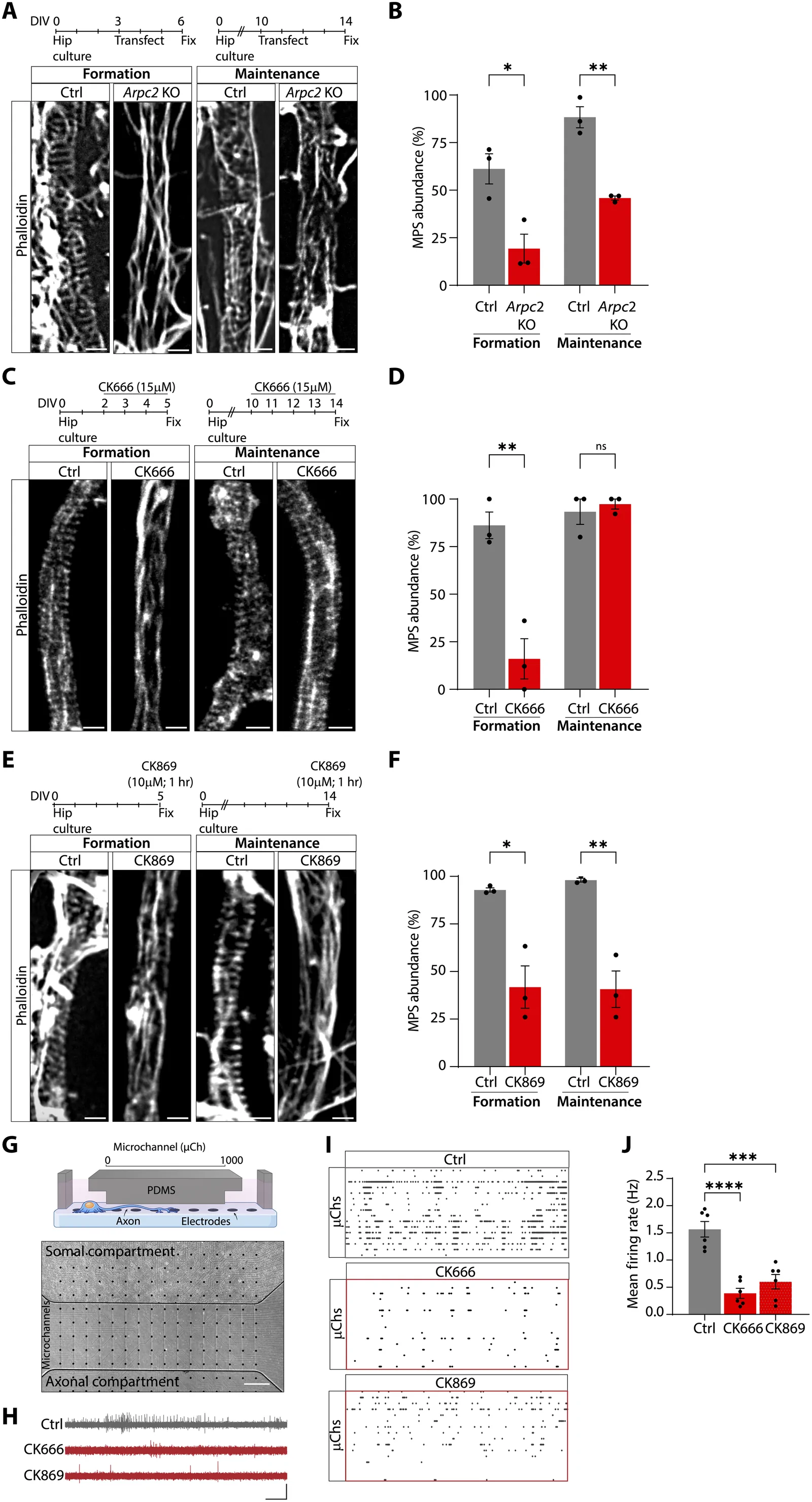
Arp2/3 is required for MPS actin ring formation and maintenance. (**A**) Timelines for CRISPR-mediated KO of *Arpc2* at early (DIV3 to DIV6) (left) and late (DIV10 to DIV14) (right) MPS development and respective Tau-STED of phalloidin-stained axonal actin rings in control (Ctrl; CRISPR targeting *Rosa26*) and *Arpc2* KO neurons at DIV6 (formation) and DIV14 (maintenance). Scale bars, 0.5 μm. (**B**) MPS abundance related to (A); *n* = 3 independent experiments (eight axons per experiment). (**C**) Timelines for CK666 treatment at early (left) or late (right) MPS assembly and representative STED of phalloidin-stained axonal actin rings in control (Ctrl; DMSO) and CK666-treated neurons at DIV5 (left) or DIV14 (right). Scale bars, 0.5 μm. (**D**) MPS abundance related to (C). *n* = 3 independent experiments (10 axons per experiment). (**E**) Timeline of CK869 treatment at DIV5 or DIV14 and respective Tau-STED of phalloidin-stained axonal actin rings in control (Ctrl; DMSO) and CK869-treated hippocampal neurons. Scale bars, 0.5 μm. hr, hours. (**F**) MPS abundance related to (E). *n* = 3 independent experiments (10 axons per experiment). (**G**) Representation of a microchannel (μCh) (top) together with phase-contrast microscopy (10× objective, mosaic) of DIV5 hippocampal neurons cultured in a μEF device (bottom). Scale bar, 200 μm. (**H**) Electrophysiological trace of 10 s of baseline activity from an electrode (within a microchannel) of control neurons (Ctrl) and neurons treated with CK666 or CK869. Vertical scale bar, 100 μV; horizontal scale bar, 1 s. (**I**) Representative raster plots of 1 min of neuronal activity for control (top), CK666-treated (middle), and CK869-treated neurons (bottom). Each row corresponds to the spike raster plot from one electrode per microchannel. (**J**) MFR related to (I); *n* = 6 independent μEFs per condition. Results are the means ± SEM; unpaired *t* test (B, D, and F) and one-way ANOVA with Tukey’s post hoc test (J); ****P ≤ 0.0001, ***P ≤ 0.001, **P ≤ 0.01, and *P ≤ 0.05; ns, nonsignificant.

To assess the role of Arp2/3 in actin ring nucleation, we disrupted *Arpc2*, a core subunit required for Arp2/3 complex stability and actin nucleation [reviewed in (*21*)] using CRISPR-Cas9 gene editing (fig. S1A). Given the low transfection efficiency of hippocampal neurons, validation of CRISPR-Cas9–mediated editing by DNA sequencing was performed in a rat cell line (fig. S1, B and C), and that by immunofluorescence was performed in transfected primary neurons (fig. S1, D and E). *Arpc2* was disrupted in primary rat hippocampal neurons either at the onset of MPS actin ring assembly (DIV3) or after the rings had formed along the entire axon shaft (DIV10), with neurons subsequently fixed at DIV6 or DIV14, respectively (Fig. 2, A and B). At this time frame, no effects were observed in neuronal polarization or axon-dendritic morphology (fig. S1, F to L). *Arpc2* knockout (KO) axons exhibited a significant reduction in MPS abundance either when *Arpc2* was edited early during its formation or later when already fully assembled throughout the axon shaft (Fig. 2, A and B). In the absence of Arp2/3, the axon shaft was enriched in long longitudinal fibers of F-actin (Fig. 2A).

To further substantiate the need of Arp2/3 for MPS actin ring formation and maintenance, we inhibited Arp2/3 activity using two small-molecule inhibitors, CK666 and CK869 (*38*). At the drug concentrations and exposure times used, the axon and dendrite length and arborization were largely preserved, except a modest difference proximal to the soma in CK666-treated neurons (fig. S1, M to S). Thus, the effects on MPS abundance were not secondary to general drug cytotoxicity. Chronic CK666 treatment from DIV2 to DIV5 resulted in severe MPS disruption (Fig. 2, C and D). In contrast, treatment with CK666 from DIV10 to DIV14 had no major impact on the MPS structure (Fig. 2, C and D). As CK666 inhibition did not recapitulate *Arpc2* deletion in later stages of in vitro neuron development, we examined the impact of CK869, which has a different mechanism of action (*38*). As with *Arpc2* disruption, acute treatment with CK869, for 1 hour, at either DIV5 or DIV14 resulted in MPS disorganization (Fig. 2, E and F). Consistent with the MPS role in regulating axonal conduction (*9*), we performed electrophysiological recordings using microelectrode arrays coupled to microfluidics (μEFs). Neurons treated with Arp2/3 inhibitors had a reduced mean firing rate (MFR) (Fig. 2, G to J), whereas the inactive analog CK689 had no effect (fig. S1T). This indicates that Arp2/3-dependent MPS disruption leads to impaired neuronal activity. Notably, different Arp2/3 isocomplexes have distinct sensitivities to CK666 and CK869 (*39*). Whereas both CK666 and CK869 inhibit actin branch formation by ARPC1A-containing isocomplexes, CK869 only inhibits those containing ARPC1B (*39*). These observations are consistent with a model in which distinct Arp2/3 isocomplexes contribute differently to MPS maturation, with ARPC1A-containing complexes potentially contributing more prominently during early stages of actin ring assembly, whereas ARPC1B-containing complexes may support MPS maintenance at later stages of neuronal development.

### ARPC1A-containing Arp2/3 isocomplexes are essential for actin ring formation

**Fig. 3.**
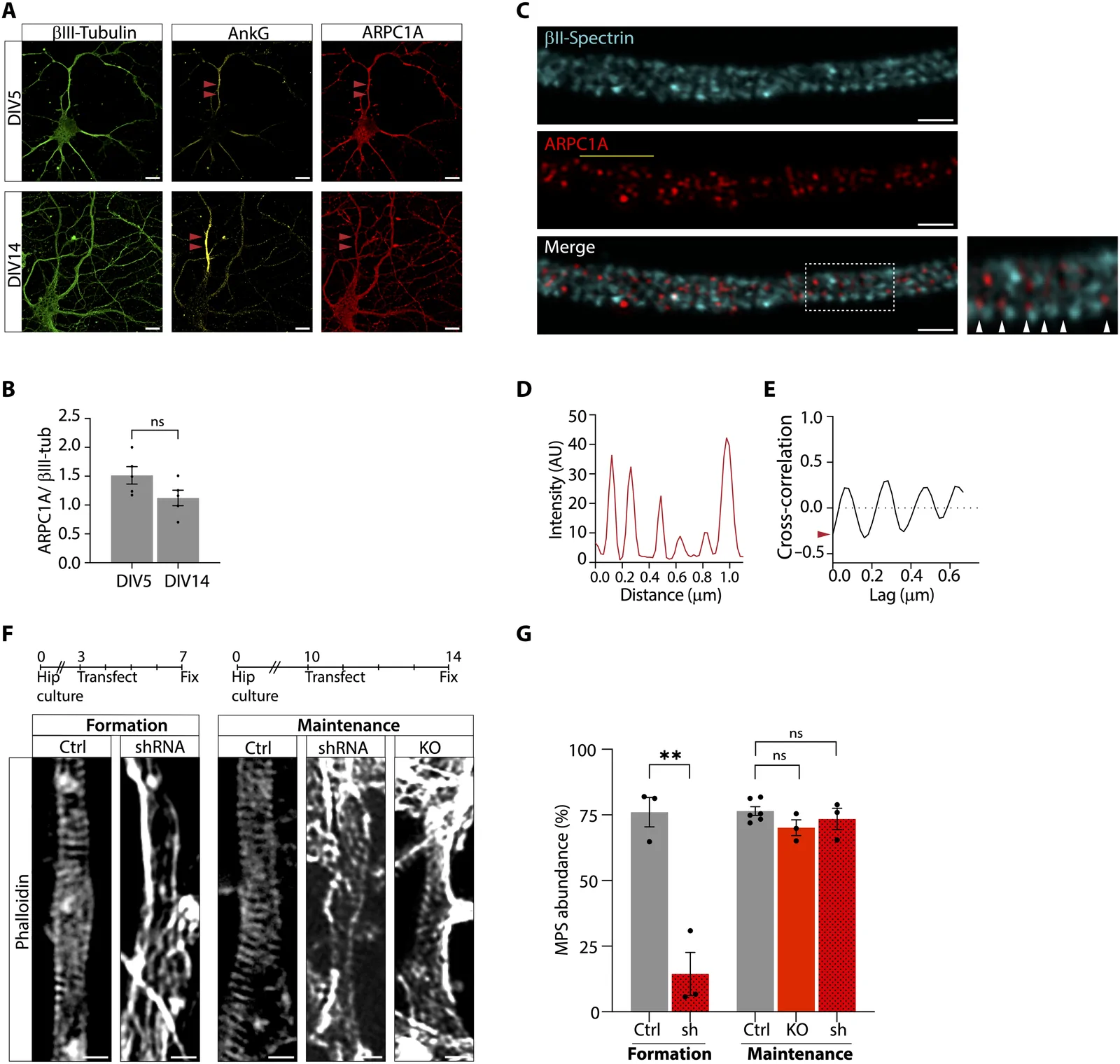
ARPC1A-containing Arp2/3 isocomplexes are essential for actin ring formation. (**A**) Representative confocal images of DIV5 (top) and DIV14 (bottom) hippocampal neurons stained for βIII-tubulin (neuronal marker), AnkG (AIS marker), and ARPC1A. Red arrowheads highlight the axon. Scale bars, 10 μm. (**B**) Quantification of axonal ARPC1A signal intensity normalized to βIII-tubulin (A) at DIV5 and DIV14. *n* = 5 independent experiments (10 neurons analyzed per experiment). (**C**) Representative two-color Tau-STED imaging of DIV5 hippocampal axons stained for βII-spectrin (cyan) and ARPC1A (red). A zoom-in of boxed region, illustrating their periodic and alternating distribution along the axon (yellow arrowheads), is shown. Scale bars, 0.5 μm. (**D**) Fluorescence intensity line profile across the yellow line within the axon in (C). (**E**) Cross-correlation curve of fluorescence intensity profiles from the boxed region shown in (C). The red arrowhead indicates the negative value at zero lag. (**F**) Experimental timeline for ARPC1A down-regulation or gene editing at early and late stages of neuron development. Phalloidin-stained Tau-STED images of control neurons (Ctrl, treated with either a scramble shRNA or a CRISPR construct targeting *Rosa26*) and of neurons where *Arpc1A* was targeted either by shRNA or edited through CRISPR (KO). Scale bars, 0.5 μm. (**G**) Quantification of MPS abundance related to (F). *n* = 3 independent experiments (eight axons analyzed per experiment). Results are shown as the means ± SEM; unpaired *t* test; **P ≤ 0.01; ns, nonsignificant.

The ARPC1 subunit that stabilizes the Arp2/3 complex and mediates interactions with nucleation-promoting factors exists as two different isoforms—ARPC1A and ARPC1B (*21*, *31*, *39*). ARPC1A was present in hippocampal neurons at DIV5 and DIV14 (Fig. 3, A and B). At DIV5, ARPC1A exhibited a periodic pattern of 175 ± 15 nm along the axon that alternated with βII-spectrin on the basis of cross-correlation analysis (Fig. 3, C to E). Notably, when *Arpc1A* was edited at DIV3 (fig. S2, A to C, G, and H), neurons had poor survival, precluding meaningful analysis at DIV7. To circumvent this, a short hairpin RNA (shRNA) targeting *Arpc1A* was used (fig. S2, D to F). Hippocampal neurons where ARPC1A was knocked down at DIV3 exhibited a marked disruption of actin ring organization, with a significant reduction in MPS abundance compared to controls (Fig. 3, F and G). Consistent with its role during early MPS assembly, *Arpc1A* knockdown significantly reduced the AIS length (fig. S2, I and J). In contrast, when *Arpc1A* was targeted after MPS assembly, either by gene editing or shRNA-mediated down-regulation, the MPS was preserved (Fig. 3, F and G). Thus, consistent with our inhibitor results, ARPC1A-containing Arp2/3 isocomplexes are essential during the early phases of MPS formation but are dispensable for actin ring maintenance.

### ARPC1B is sufficient to sustain MPS actin rings in mature neurons

**Fig. 4.**
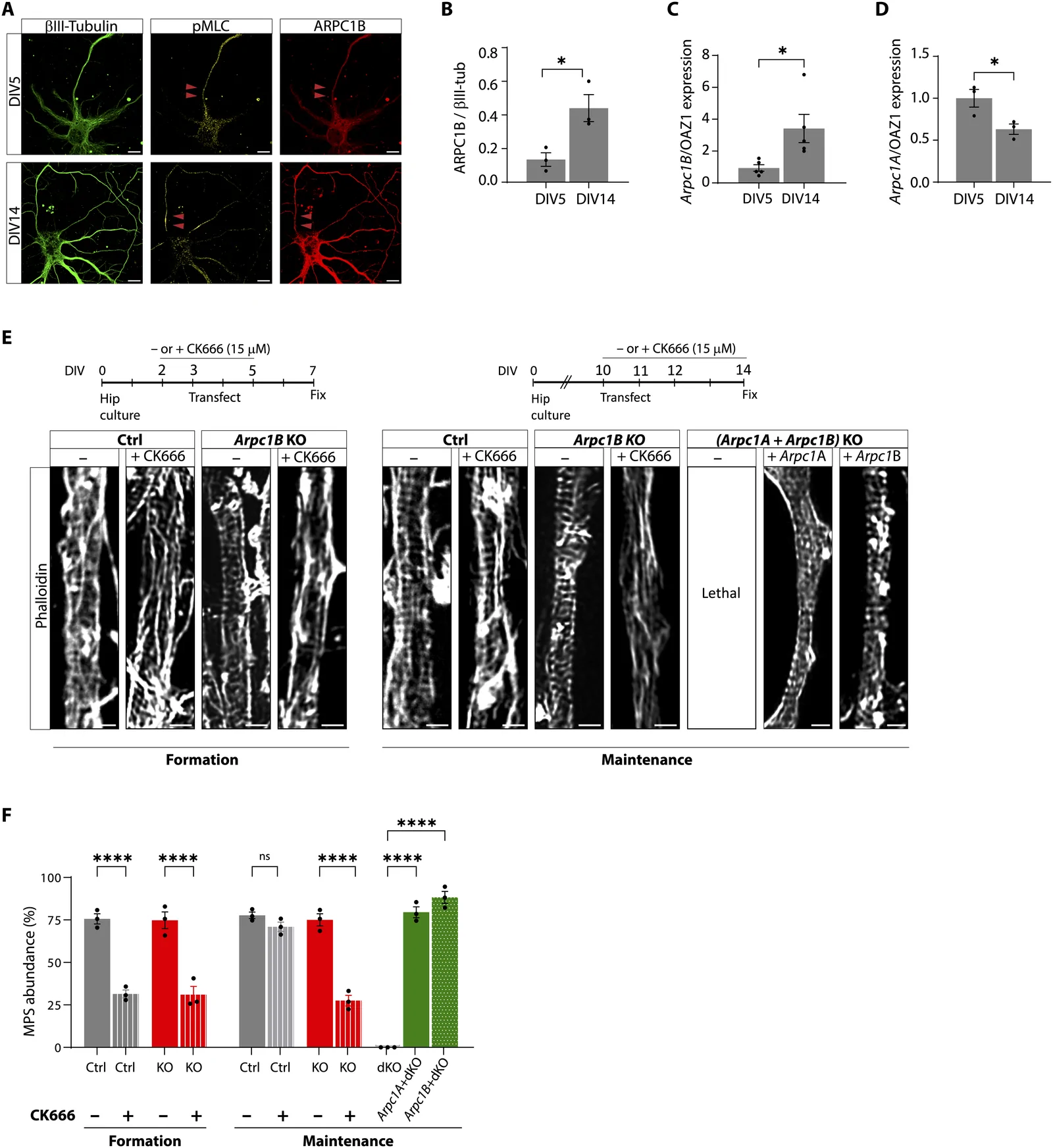
ARPC1B-containing Arp2/3 isocomplexes are sufficient for actin ring maintenance. (**A**) Representative confocal images of DIV5 (top) and DIV14 (bottom) hippocampal neurons stained for βIII-tubulin (neuronal marker), pMLC (AIS marker), and ARPC1B. Red arrowheads highlight the axon. Scale bars, 10 μm. (**B**) Quantification of ARPC1B signal intensity normalized to βIII-tubulin in axons at DIV5 and DIV14. *n* = 3 independent experiments (10 neurons analyzed per experiment). (**C**) qPCR analysis of *Arpc1B* expression levels, normalized to *OAZ1* relative to DIV5. *n* = 5 independent experiments (triplicates per experiment). (**D**) qPCR analysis of *Arpc1A* expression levels, normalized to *OAZ1* relative to DIV5. *n* = 3 independent experiments (triplicates per experiment). (**E**) Experimental timeline for CRISPR editing of *Arpc1B* and CK666 treatment at early and late stages of MPS development and representative Tau-STED images of hippocampal axons stained with phalloidin in control (Ctrl; CRISPR construct targeting *Rosa26*); *Arpc1B* edited (KO), with (+) or without (−) CK666 treatment; and *Arpc1A* and *Arpc1B* dKO, with or without *Arpc1A* or *Arpc1B* rescue. Scale bars, 0.5 μm. (**F**) Quantification of MPS abundance related to (E). *n* = 3 independent experiments (KO and dKO; 10 and 8 neurons were analyzed per experiment, respectively). Results are shown as the means ± SEM; unpaired *t* test (B to D) and one-way ANOVA with Tukey’s post hoc test (F); ****P ≤ 0.0001 and *P ≤ 0.05; ns, nonsignificant.

While ARPC1A-containing Arp2/3 isocomplexes are essential for MPS formation, our data using chemical inhibition support the notion that ARPC1B-containing Arp2/3 isocomplexes are important for MPS maintenance during later developmental stages. Supporting this notion, *Arpc1B* mRNA and protein levels increased from DIV5 to DIV14 in contrast to *Arpc1A* (Fig. 4, A to D). However, when *Arpc1B*-deficient hippocampal neurons were generated by CRISPR-Cas9 KO targeting (fig. S3, A to E), no significant disruption of the MPS was observed at either DIV7 or DIV14 (Fig. 4, E and F), and the AIS length was unaffected (fig. S2, I and J). This suggests that ARPC1B is not required for MPS formation or maintenance. This led us to hypothesize that ARPC1A might compensate for the loss of ARPC1B. To test this possibility, we treated *Arpc1B* KO neurons with CK666, as it selectively targets ARPC1A-containing Arp2/3 isocomplexes (*39*). In the presence of CK666, MPS actin ring periodicity in *Arpc1B* KO neurons was disrupted at both DIV7 and DIV14 (Fig. 4, E and F). This result phenocopies deletion of *Arpc2* or treatment with CK869 (Fig. 2, A to F). To further confirm that ARPC1A and ARPC1B are functionally redundant, we performed a CRISPR-Cas9 double KO (dKO) for *Arpc1A* and *Arpc1B* (Fig. 4, E and F). Combined depletion of both isoforms resulted in a severe loss of neuronal viability (Fig. 4, E and F). This effect and the MPS organization were rescued by the overexpression of either *Arpc1A* or *Arpc1B* in the dKO, further showing their functional redundancy (Fig. 4, E and F). Collectively, these data indicate that ARPC1B is sufficient to sustain actin rings at later stages of MPS development, while ARPC1A can functionally buffer in its absence.

### ARPC5 is necessary for the early MPS assembly

**Fig. 5.**
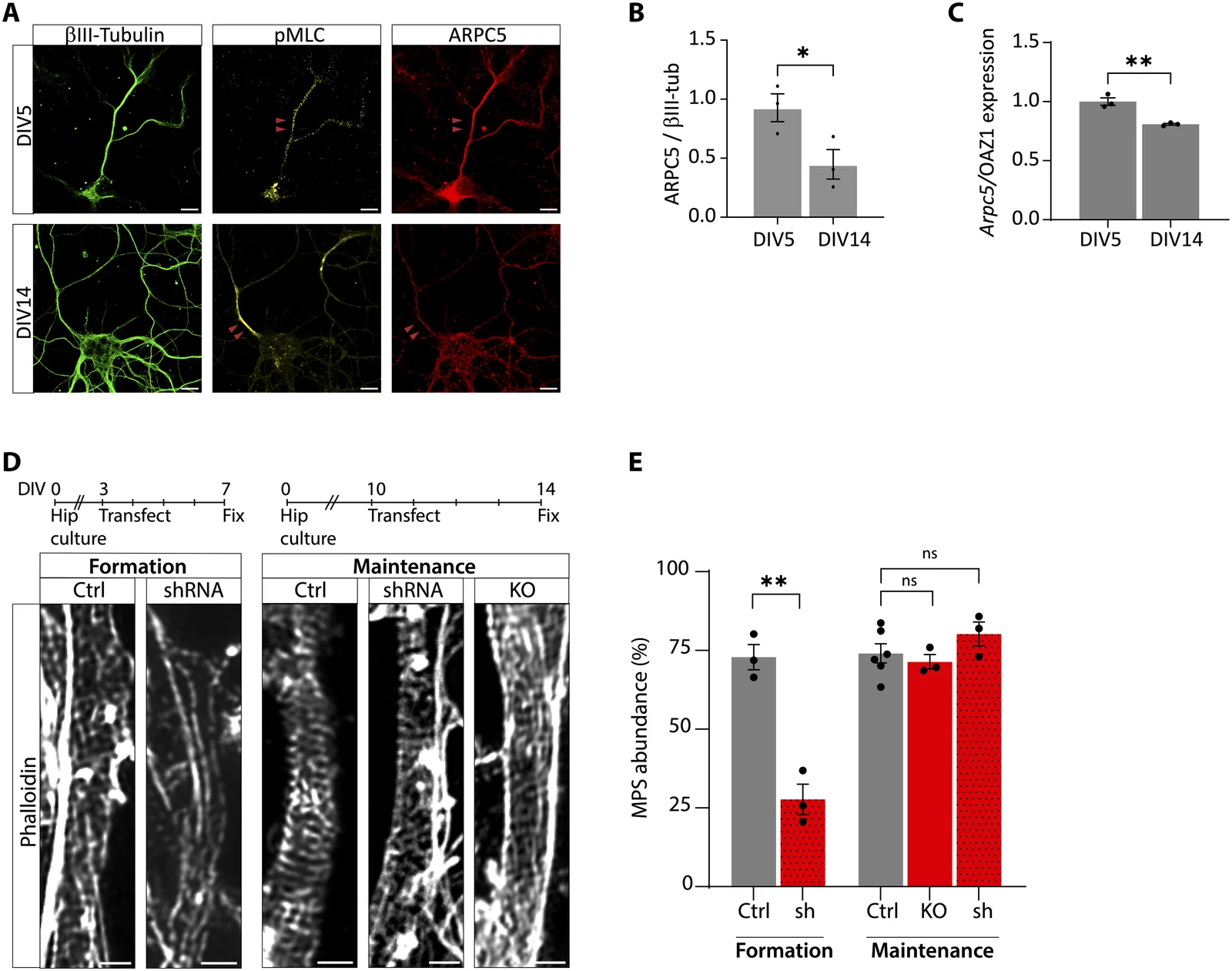
ARPC5-containing isocomplexes are essential for the MPS actin ring formation. (**A**) Representative confocal images of DIV5 (top) and DIV14 (bottom) hippocampal neurons stained for βIII-tubulin (neuronal marker), pMLC (AIS marker), and ARPC5. Red arrowheads highlight the axon. Scale bars, 10 μm. (**B**) Quantification of ARPC5 signal intensity normalized to βIII-tubulin in axons at DIV5 and DIV14. *n* = 3 independent experiments (12 neurons analyzed per experiment). (**C**) qPCR analysis of *Arpc5* expression levels, normalized to *OAZ1* relative to DIV5. *n* = 3 independent experiments. (**D**) Experimental timeline for shRNA- and/or CRISPR-Cas9–mediated editing of *Arpc5* at early (left) and late (right) stages of MPS formation and respective Tau-STED images of phalloidin-stained axonal actin rings in control (CRISPR construct targeting *Rosa26*) and *Arpc5* KO neurons at DIV7 and DIV14. Scale bars, 0.5 μm. (**E**) Quantification of MPS abundance from (D). *n* = 3 independent experiments (10 axons analyzed per experiment). Results are shown as the means ± SEM; unpaired *t* test; **P ≤ 0.01 and *P ≤ 0.05; ns, nonsignificant.

The ARPC5- and ARPC5L-containing Arp2/3 isocomplexes have been differentially implicated in modulating actin dynamics in non-neuronal systems, such as HeLa cells, T cells, and macrophages (*31*, *35*, *36*, *39*–*41*). Given this, we investigated whether the functional specialization seen for ARPC1 isocomplexes in MPS formation and maintenance extends to other ARPC5 isoforms. ARPC5 mRNA and axonal protein levels were more prominent at DIV5, supporting a developmental reduction in ARPC5 expression as neurons mature (Fig. 5, A to C). Targeting the *Arpc5* gene with CRISPR-Cas9 at early DIVs compromised neuronal survival (fig. S4, A to C, G, and H). In contrast, shRNA-mediated knockdown of ARPC5 resulted in a pronounced disruption of actin ring organization (fig. S4, D to F, and Fig. 5, D and E) and in a reduction of AIS length (fig. S2, I and J). When *Arpc5* was targeted at DIV10 using shRNA or CRISPR-Cas9 gene editing, the MPS organization was preserved (Fig. 5, D and E). This indicates that ARPC5-containing Arp2/3 isocomplexes are essential during MPS assembly but are not needed for maintaining the actin ring architecture in mature neurons.

### ARPC5L is essential for maintaining the mature MPS and for neuronal survival and function

**Fig. 6.**
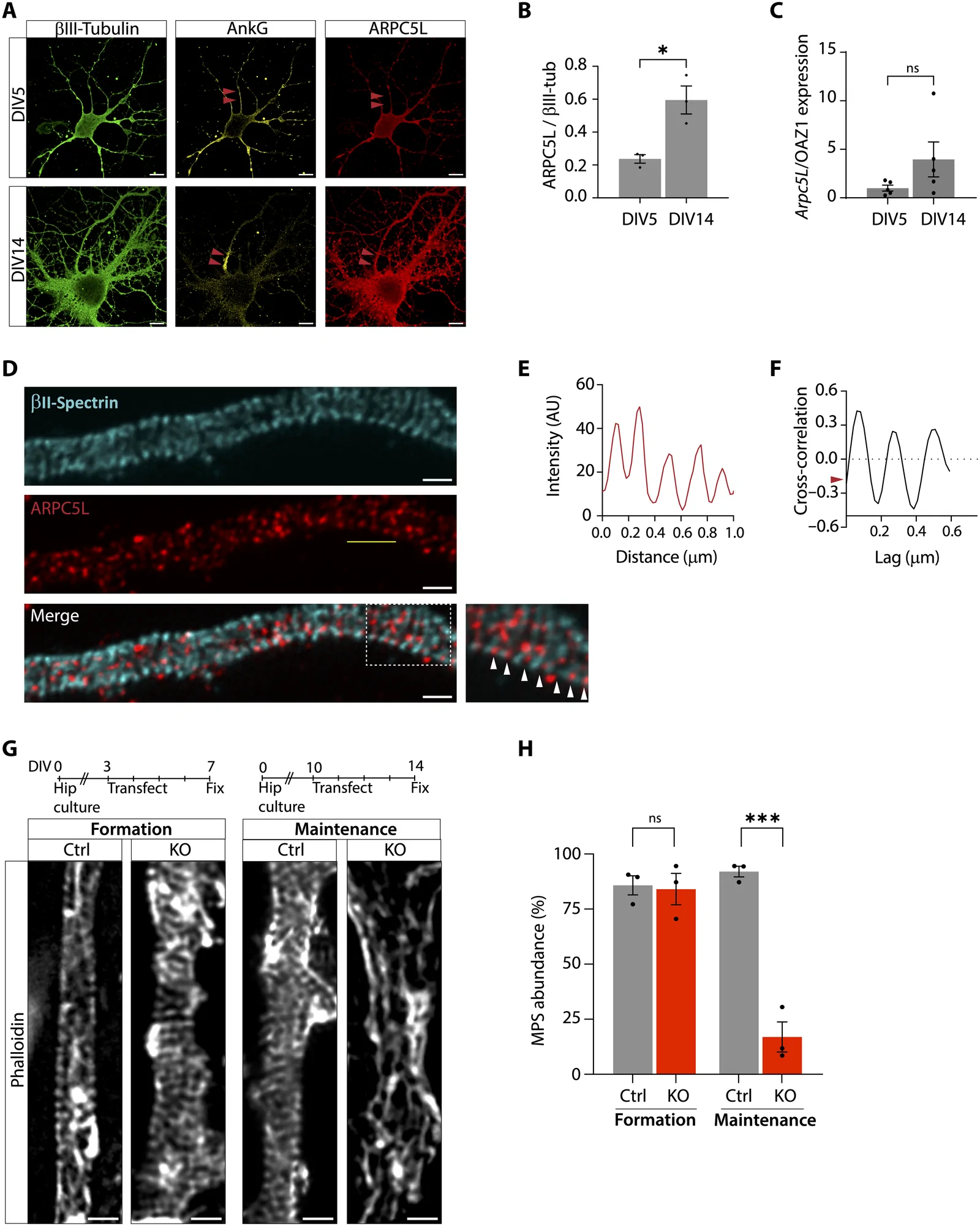
ARPC5L-containing isocomplexes are essential for the MPS actin ring maintenance. (**A**) Representative confocal images of DIV5 (top) and DIV14 (bottom) hippocampal neurons stained for βIII-tubulin (neuronal marker), AnkG (AIS marker), and ARPC5L. Red arrowheads highlight the axon. Scale bars, 10 μm. (**B**) Quantification of ARPC5L signal intensity normalized to βIII-tubulin in axons at DIV5 and DIV14*. n* = 3 independent experiments (10 neurons analyzed per experiment). (**C**) qPCR analysis of *Arpc5L* expression levels, normalized to *OAZ1* relative to DIV5. *n* = 5 independent experiments (triplicates per experiment). (**D**) Two-color Tau-STED imaging of DIV14 hippocampal axons stained for βII-spectrin (cyan) and ARPC5L (red). A zoom-in of the boxed region is provided, illustrating their periodic and alternating distribution along the axon (white arrowheads). Scale bars, 0.5 μm. (**E**) Fluorescence intensity line profile across the yellow line within the axon in (D). (**F**) Cross-correlation curve of fluorescence intensity profiles from the region shown in (D). The red arrowhead indicates the negative value at zero lag. (**G**) Experimental timeline for CRISPR-mediated KO of *Arpc5L* at early and late stages of MPS development and Tau-STED images of phalloidin-stained axonal actin rings in control (Ctrl; CRISPR construct targeting *Rosa26*) and *Arpc5L* KO neurons edited during MPS formation or maintenance. Scale bars, 0.5 μm. (**H**) Quantification of MPS abundance relative to (G). *n* = 3 independent experiments (eight axons analyzed per experiment). Results are shown as the means ± SEM; unpaired *t* test; ***P ≤ 0.001 and *P ≤ 0.05; ns, nonsignificant.

We then examined ARPC5L, which, in contrast to ARPC5, had increased levels at DIV14 (Fig. 6, A to C). Two-color STED nanoscopy showed that ARPC5L has a periodic distribution of 183 ± 25 nm that alternates with βII-spectrin (Fig. 6, D to F). Editing of the *Arpc5L* gene (fig. S5, A to E) showed that in contrast to ARPC5 (Fig. 5), ARPC5L-deficient neurons transfected at DIV3 remained viable, with a well-organized MPS (Fig. 6, G and H), and the AIS length was unaffected (fig. S2, I and J). However, editing the *Arpc5L* gene at DIV10 disrupted actin rings at DIV14 (Fig. 6, G and H), supporting the essential role of ARPC5L in sustaining the MPS in mature neurons.

**Fig. 7.**
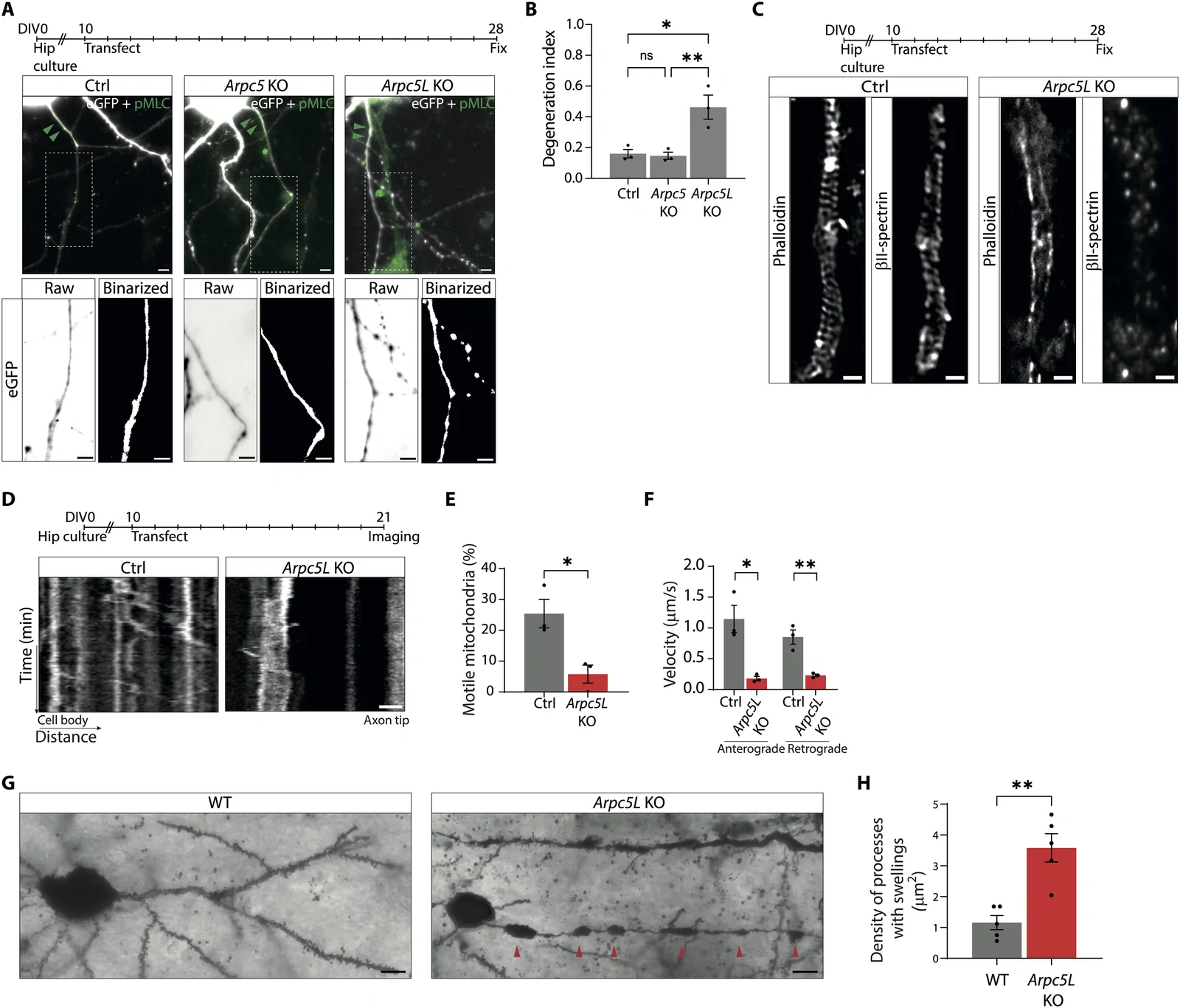
ARPC5L is essential for maintaining the mature MPS and for neuronal survival and function. (**A**) Timeline and representative fluorescence images of hippocampal neurons transfected at DIV10 with CRISPR constructs targeting the *Rosa26* locus (control), *Arpc5*, or *Arpc5L*. Neurons were fixed at DIV28 and immunostained for pMLC to label the AIS (green). eGFP fluorescence marks transfected neurons (gray). Boxed regions indicate the axon segment analyzed for degeneration. Raw (left) and binarized (right) images of the axon segment [boxed area in (A)] used for quantification of axon degeneration. Scale bars, 5 μm. (**B**) Quantification of the axon degeneration across conditions. *n* = 3 independent experiments (10 axons per condition were analyzed per experiment). Data are shown as the means ± SEM. One-way ANOVA followed by Tukey’s post hoc test; ***P < 0.001, **P < 0.01, and *P < 0.05. (**C**) Timeline and representative images of axons from hippocampal neurons transfected at DIV10 with CRISPR constructs targeting either the Rosa*26* locus (control) or *Arpc5L* and stained with phalloidin or βII-spectrin. Scale bars, 0.5 μm. (**D**) Timeline and representative kymograms of mitochondrial transport in control and *Arpc5L*-depleted neurons. Neurons were transfected at DIV10 and live imaged at DIV21. Kymograms illustrate mitochondrial movement along axons. Scale bar, 25 μm. (**E** and **F**) Quantification of the percentage of motile mitochondria (E) and anterograde and retrograde mitochondrial velocities (F). *n* = 3 independent experiments (eight axons analyzed per experiment); unpaired *t* test; **P ≤ 0.01 and *P ≤ 0.05; ns, nonsignificant. (**G**) Representative images of Golgi-stained cortical neurons from WT and *Arpc5L* KO mice at P30. Scale bars, 10 μm. (**H**) Quantification related to (G). *n* = 5 brains were analyzed per genotype. Data are shown as the means ± SEM; unpaired *t* test; **P ≤ 0.01 and *P ≤ 0.05; ns, nonsignificant.

Accordingly, *Arpc5L* depletion in fully matured neurons (DIV10) led to overt axon fragmentation (analyzed at DIV28), whereas *Arpc5* depletion failed to induce a similar effect (Fig. 7, A and B). These data further show that ARPC5L is critical to maintaining the MPS in mature neurons, while ARPC5 is not required for MPS maintenance or neuron survival. Consistent with the role of the MPS in the early steps of degeneration (*16*, *17*), *Arpc5L*-depleted neurons displayed a marked disruption of the MPS, lacking periodic actin and βII-spectrin organization (Fig. 7C). Severe axonal transport defects, arising either from MPS regulation of axonal transport (*12*) or from ongoing degeneration, were also observed in *Arpc5L*-depleted neurons, which exhibited a marked reduction in the number of motile mitochondria and their velocities (Fig. 7, D to F). To further validate the in vitro data, we investigated whether the absence of *Arpc5L* in vivo in *Arpc5L* KO mice was sufficient to cause neuronal defects. In *Arpc5L* KO mice, Golgi staining revealed an increased number of neurites with swellings when compared with wild-type (WT) mice, consistent with axonal stress and degeneration (Fig. 7, G and H). These data further support the notion that ARPC5 and ARPC5L are not functionally interchangeable in neuronal biology and that ARPC5L is critical for maintaining neuronal health.

## DISCUSSION

Collectively, our findings support a model in which Arp2/3 is a component of the MPS, allowing branched actin filaments to contribute to its nanoarchitecture. On the basis of early superresolution imaging studies and the periodic localization of adducin, MPS actin rings were initially proposed to consist of multiple short and capped actin filaments (*3*). In contrast, more recent work suggested that MPS actin rings consist of two long, intertwined filaments, implying a more continuous and potentially more stable arrangement (*18*). By identifying Arp2/3 as an essential component in the formation and maintenance of the MPS, we propose that beyond the contribution of linear actin filaments, branched actin networks nucleated by Arp2/3 are part of the MPS architecture. In other cellular contexts, higher-order actin structures such as the actin cortex [reviewed in (*42*)], adherens junctions (*43*), and the actin rings formed during epithelial wound closure (*44*) are formed through the coordinated activity of both linear and branched actin filaments. In the case of cytokinesis, the contractile ring itself is primarily composed of formin-generated linear actin filaments (*45*), while Arp2/3-dependent networks are typically adjacent (*45*) but yet contribute to the proper assembly and dynamics of the contractile apparatus (*46*). The cooperation between Arp2/3-mediated branching and formin-mediated elongation may represent a common principle in the formation and regulation of ring-like actin structures in different biological contexts. In line with this, actin rings in *Drosophila* axons depend on Arp2/3 and formins (*13*), and in hippocampal neurons, the formin inhibitor SMIFH2 compromises the periodic MPS arrangement (*47*) and dynamics (*48*). These findings further support the notion that in neurons, the MPS might be composed of a higher-order actin nanoarchitecture where both branched and linear actin filaments coexist.

Our data further support the notion that a switch in Arp2/3 isocomplex levels orchestrates MPS formation and maturation. While isocomplexes with ARPC1A and ARPC5 are functionally relevant in regulating the initial MPS actin ring assembly, a transition toward ARPC1B- and ARPC5L-containing Arp2/3 isocomplexes occurs in the mature MPS, which is essential for its structural maintenance. This isoform-specific shift may reflect a functional specification of the Arp2/3 complex to support the transition from dynamic actin ring assembly to a more structurally stable MPS architecture. This would mirror findings in other cellular systems. In non-neuronal cells such as macrophages (*39*), fibroblasts (*49*), and T cells (*41*), ARPC1A/ARPC5-containing complexes have been associated with more dynamic events. This behavior is likely due to intrinsic properties of different Arp2/3 isocomplexes as ARPC1A- and ARPC5-containing Arp2/3 have reduced actin nucleation activity and generate branched actin networks with shorter lifetimes when compared to their ARPC1B/ARPC5L counterparts (*31*). Thus, switching from ARPC1A/ARPC5 to ARPC1B/ARPC5L may reflect a general mechanism by which cells temporally regulate the balance between actin remodeling and stabilization in complex structures. Recently, the MPS was shown to be dynamic, undergoing constitutive cycles of local disassembly and reassembly (*48*). This reinforces the notion that the MPS can be fine-tuned to adapt to the needs of the cell, which will require remodeling of actin rings. At early developmental stages (DIV2 to DIV5), the emerging MPS is more sensitive to disruption by actin-depolymerizing agents such as latrunculin A and cytochalasin D, indicating an inherently less stable and more dynamic actin-spectrin lattice (*4*, *13*). At later developmental stages (around DIV10), FRAP (fluorescence recovery after photobleaching) analyses of βII-spectrin-green fluorescent protein (GFP) in hippocampal neurons revealed that there is more limited spectrin turnover, indicating that the MPS is more stable (*3*). This developmental trajectory—from a labile to more stable structure—could be assisted by the switch in Arp2/3 isocomplex composition. In addition to their role in MPS biology, distinct Arp2/3 isocomplexes may participate in other aspects of neuron biology. As an example, Arp2/3 nucleates dynamic actin patches at the AIS, which regulate polarized cargo trafficking and contribute to the maintenance of axonal identity (*50*). Thus, some of the phenotypes observed following depletion or inhibition of different Arp2/3 isocomplexes could also reflect alterations in AIS-associated actin patches.

The Arp2/3 isoform switch described here is not unprecedented in neuronal biology. Similar developmentally regulated transitions have been observed for other cytoskeletal proteins, notably the well-characterized switch from βIVΣ1 to βIVΣ6 spectrin isoforms, which occurs at the AIS and nodes of Ranvier (*51*). In this case, alternative splicing of the *SPTBN4* gene underlies βIVΣ1 isoform prevalence in early development and its progressive replacement by βIVΣ6 in mature neurons, reflecting a transition from cytoskeletal assembly to structural maintenance.

In summary, we propose that in axons, Arp2/3 isocomplexes have specific temporal functions. In the initial phases of MPS assembly, actin rings comprise branched filaments with higher turnover generated by ARPC1A/ARPC5-containing Arp2/3 isocomplexes, whereas in mature axons, ARPC1B/ARPC5L-containing complexes support the formation of a network with more stable branched filaments. Overall, our data broaden the understanding of cytoskeletal specialization in neurons and open avenues for dissecting how actin network diversity underpins neuronal function.

## MATERIALS AND METHODS

### Animals

Wistar rats were handled according to both the National Decree Law no. 113-2013 and the European Union Directive 2010/63/EU regulations (project license number 022864/2020-12-31). Neuron-specific *Arpc5L* KO mouse strains were developed at The Francis Crick Institute and were previously reported (*36*) by crossing *Arpc5L* floxed mice (*35*) with NEX-cre mice (a gift from K. Nave, Max Planck Institute for Multidisciplinary Sciences) (*52*); animals of both sexes were used at postnatal day 30 (P30). All strains were maintained on a C57BL/6J background. Mouse studies were designed under the up-to-date national and institutional guidelines and approved by the UK Home Office and Animal Welfare and Ethical Review Body of The Francis Crick Institute under project license number PP0792028. This study followed the ARRIVE (Animal Research: Reporting of In Vivo Experiments) guidelines for reporting animal research. All animals were maintained with ad libitum access to water/food and kept on a 12-hour light/dark cycle under controlled temperature and humidity*.*

### Hippocampal neuron culture

Wistar rat hippocampal neuron cultures were established as described previously (*53*). Briefly, the hippocampus of each individual embryonic day 18 embryo was digested for 15 min in 0.06% trypsin (T4799, Sigma-Aldrich) and mechanically dissociated into a suspension. Either 15,000 cells per coverslip (for the analysis of endogenous protein levels) or 50,000 cells per coverslip (in the case of transfections) were plated onto poly-l-lysine hydrobromide (50 μg/ml; Sigma-Aldrich)–precoated, plasma-cleaned (Tergeo Plasma Cleaner), 1.5H glass, 13-mm rounded coverslips (MARI0117530, Marienfeld) in 24-well plates (Nunc). For quantitative polymerase chain reaction (qPCR) and STORM/DNA-PAINT imaging, 100,000 cells per coverslip or 50,000 cells were plated on 1.5H, 18-mm round glass coverslips (MARI0107032, Marienfeld) placed in six-well plates (Nunc). Neurons were cultured in Neurobasal medium (Thermo Fisher Scientific) supplemented with 2× B-27 (Thermo Fisher Scientific), 1% penicillin/streptomycin (Thermo Fisher Scientific), and 2 mM l-glutamine (Thermo Fisher Scientific).

### Cell line

Rat-2 cells (CRL-1764, American Type Culture Collection), a rat fibroblast cell line, were cultured in high-glucose Dulbecco’s modified Eagle’s medium with 2 mM l-glutamine and 1 mM sodium pyruvate, supplemented with 10% fetal bovine serum (FBS) and 1% penicillin/streptomycin. Rat-2 cells were used for validation of CRISPR and shRNA, as detailed below.

### Immunolabeling

Unless stated otherwise, primary hippocampal neurons were fixed with 4% (w/v) paraformaldehyde (PFA) in phosphate-buffered saline (PBS; pH 7.4) for 15 min at room temperature. Fixed cells were permeabilized with 0.1% (v/v) Triton X-100 (in PBS) for 5 min, and the autofluorescence was quenched with 0.2 M ammonium chloride (in H_2_O) and blocked in 5% FBS in PBS for 1 hour. Primary antibodies recognizing the rat ortholog of the target protein were incubated overnight at 4°C. The following primary antibodies diluted in blocking buffer were used: polyclonal guinea pig anti–βIII-tubulin (Synaptic Systems, 302 304, 1:200), monoclonal mouse anti–Ankyrin-G (Santa Cruz Biotechnology, sc-12719, 1:200), polyclonal rabbit anti-ARPC2 (Sigma-Aldrich, HPA008352, 1:50), polyclonal rabbit anti-pMLC2 Thr^18^/Ser^19^ (Cell Signaling, 1673674S, 1:50), monoclonal mouse anti-cortactin (Upstate, 05-180, 1:50), monoclonal mouse anti–βII-spectrin (BD Transduction, 612562, 1:200), polyclonal rabbit anti-ARPC1A (Sigma-Aldrich, HPA004334-100UL, 1:50, or Proteintech, 17538-1-AP, 1:100), monoclonal mouse anti-ARPC1B (Santa Cruz, sc-65168, 1:50), monoclonal mouse anti-ARPC1B (Proteintech, 67320-1-Ig, 1:100), monoclonal mouse anti-ARPC5 (Santa Cruz, sc-166760, 1:50), and monoclonal rabbit anti-ARPC5L (Abcam, EPR10274, 1:50). After three 5-min washes in PBS, incubation with a secondary antibody was performed for 1 hour at room temperature. For confocal and STED microscopy, the following secondary antibodies were used: polyclonal donkey anti–guinea pig Alexa Fluor 488 (Jackson ImmunoResearch, 706-545-148, 1:500), polyclonal goat anti-mouse STAR 580 (Abberior GmbH, ST580-1001-500UG, 1:200), polyclonal goat anti-rabbit STAR 635P (Abberior GmbH, ST635P-1002-500UG, 1:200), polyclonal goat anti-rabbit STAR 580 (Abberior GmbH, ST580-1002-500UG, 1:200), polyclonal goat anti-mouse STAR 635P (Abberior GmbH, ST635P-1001-500UG, 1:200), polyclonal donkey anti-mouse Alexa Fluor 647 (Jackson ImmunoResearch, 715-605-150, 1:1000), and polyclonal donkey anti-rabbit Alexa Fluor 594 (Thermo Fisher Scientific, A21207, 1:1000). For actin staining, 0.3 μM phalloidin 635P (Abberior GmbH, ST635-0100-20UG, in PBS) was used. Phalloidin was incubated in PBS for 1 hour at 37°C after the secondary antibody incubation. Coverslips were washed three times in PBS for 5 min, mounted in 90% glycerol [with 0.5% *N*-propyl gallate in 20 mM Tris (pH 8.0)], and sealed with nail varnish. To quantify the expression levels of ARPC2, cortactin, ARPC1A, ARPC5, and ARPC5L, we measured the fluorescence intensity in axons and dendrites of DIV5 and DIV14 primary hippocampal neurons. Neurons were immunostained for each target protein and colabeled with βIII-tubulin. Using Fiji/ImageJ, segments of axons (10 to 20 μm in length) were manually selected with the segmented line or freehand selection tool, and mean fluorescence intensity was measured for each marker within the selected region. The mean intensity of each protein was further normalized by the mean intensity of βIII-tubulin signal in the corresponding region. For every condition, *n* = 3 independent experiments were performed, each with 10 to 20 neurons.

### Quantitative PCR of Arp2/3 isoforms

Primary hippocampal neurons were plated at a density of 100,000 cells per well in six-well plates, and RNA was extracted at either DIV5 or DIV14. Total RNA was isolated using the NZY Total RNA Isolation Kit (MB13402). A total of 200 ng of RNA from each sample was used for first-strand cDNA synthesis using the NZY First-Strand cDNA Synthesis Kit (MB125), according to the manufacturer’s instructions. SYBR-green qPCR (CFX384 Touch Real-Time PCR Detection System, Bio-Rad) was performed using specific primers, designed using Beacon designer software (Biosoft). The following primers were used: *Arpc1A*: 5′-CTCATCTCTGTCTGCTACT-3′, forward; 5′-GCTTCTCATCCACCTCTT-3′, reverse; *Arpc1B*: 5′-TTGAGCAGGAGAATGACT-3′, forward; 5′-CTTGATATAGGCAGAGAAGA-3′, reverse; *Arpc*5: 5′-GCAGCATCGTCTTGAAGGT-3′, forward; 5′-CTCCTCCAGCAGCAAGTG-3′, reverse; *Arpc*5*L*: 5′-CGGAACTCTCCAATCAACACCAA-3′, forward; 5′-CTGCTCAATCTCGCTGCTCTT-3′, reverse. The fold change in gene expression was calculated using the ΔΔCt relative expression method (Livak method), and primers for ornithine decarboxylase antizyme 1 (*OAZ1*) (5′-TGCAGCAGCGAGAGTTCTAGG-3′, forward; 5′-CCGGACCCAGGTTACTACAG-3′, reverse) were used as the endogenous control. For *Arpc1A* and *Arpc*5, *n =* 3 independent experiments were performed each with triplicates. For *Arpc1B* and *Arpc5L*, *n =* 5 independent experiments were done each with triplicates.

### Arp2/3 pharmacological inhibition

To inhibit Arp2/3 complex activity, hippocampal neurons were treated with the small-molecule inhibitor CK666 (15 μM; Tocris Bioscience) or CK869 (10 μM; Sigma-Aldrich), and the inactive analog CK689 (15 μM; Merck Life Science, LTU) was used as a negative control. Drugs were titrated to select conditions that did not compromise neuronal polarization and viability. CK666 was applied chronically from DIV2 to DIV5, or DIV10 to DIV14, to assess its effects during actin ring formation and maturation. CK869 was applied acutely at DIV5 or DIV14 for 1 hour, as chronic treatment resulted in substantial cell death, to allow inhibition at the corresponding developmental stages. In all conditions, dimethyl sulfoxide (DMSO) was used as a vehicle at the same final concentration as in the drug-treated samples. For CK666 treatment in *Arpc1B* KO experiments, the inhibitor was added from DIV2 to DIV5, or from DIV10 to DIV14, and transfection was performed on DIV3 or DIV10. *n* = 3 independent experiments were performed, and 10 neurons were analyzed in each.

### Neurite outgrowth and Sholl analysis

For the analysis of neurite outgrowth, guinea pig polyclonal anti–βIII-tubulin (Synaptic Systems, 302304, 1:200) was used, followed by donkey anti-guinea pig Alexa Fluor 488 (Jackson ImmunoResearch, 1:1000), and cells were visualized in a Leica DMI 6000B with an ORCA-8 Flash4.0 V2 C11440-22CU digital camera and Leica Application Suite Advanced 9 Fluorescence (LAS AF) software. Quantifications were performed exclusively on transfected [enhanced GFP (eGFP)–positive] neurons, which, given their low density, allowed us to trace axonal and dendritic arbors. Total axon and dendritic length were traced manually with the NeuronJ plug-in for Fiji/ImageJ (*54*). *n* = 3 independent experiments, each with 10 to 20 neurons, were analyzed for every condition. For branching analysis, neuronal reconstruction and quantification were performed using Imaris software (version 10.2, Bitplane, Oxford Instruments). The soma of each neuron was manually outlined and set as the origin point for tracing, and the “Filament” tool was used to reconstruct all primary and secondary neurites. Neurite branching complexity was quantified using Sholl analysis, applying concentric circles with increasing radii of 25 μm from the soma to the distal tips of neurites. Parameters extracted included the number of intersections per radius. *n* = 3 independent experiments each with 30 neurons were analyzed for every condition.

### shRNA-mediated down-regulation of Arp2/3 isoforms

To knock down the expression of *Arpc1A* and *Arpc5*, specific shRNAs (VectorBuilder) were used: for *Arpc1A*, 5′-CATCACTCAAGTGTCTATTTA-3′ and, for *Arpc5*, 5′-GGACCACACTTCCCTTATAAA-3′. A shRNA scramble plasmid (encoding eGFP and puromycin resistance; VectorBuilder) was used as a control. At DIV3 or DIV7, hippocampal neurons were transfected with specific shRNA plasmids (encoding eGFP and puromycin resistance; 0.5 μg per well in a 24-well plate) using Lipofectamine 2000 (Invitrogen) according to the manufacturer’s instructions. Briefly, DNA (0.5 μg of scramble or shRNA) and Lipofectamine 2000 (2 μl per well) were each diluted in 50 μl of Neurobasal medium (without supplements), combined, and incubated for 15 min to allow complex formation. The transfection mixture was added dropwise to the wells and incubated for 1 hour before being replaced with fresh culture medium. Cells were fixed at DIV7 or DIV14. In all conditions, transfected (eGFP-positive) and nontransfected (eGFP-negative) cells in the same well were used for analysis.

Given that transfection of hippocampal neurons has a low efficiency, to validate shRNA knockdown efficacy, Rat-2 cells were seeded at a density of 50,000 cells per well in 24-well plates and transfected using the same Lipofectamine 2000 protocol as described above, except that the transfection mixture was incubated with the cells for 2 hours before the medium was replaced. After 48 hours, eGFP-positive cells were sorted by fluorescence-activated cell sorting (BD FACSAria Fusion) and grown in puromycin-containing medium (1.5 μg/ml). Total RNA was subsequently extracted 14 days later using the NZYtech Total RNA Isolation kit according to the manufacturer’s instructions. RNA concentration and purity were assessed using a NanoDrop spectrophotometer. For cDNA synthesis, 0.5 μg of total RNA was reverse transcribed using the NZYtech First-Strand cDNA Synthesis Kit, following the manufacturer’s protocol. Reverse transcription PCR was performed using specific primers: *Arpc1A* [5′-CGATGACCGTGGCTGTTTGA-3′, forward; 5′-GTACCAGCAACTCCCACTC-3′, reverse; 147–base pair (bp) amplicon] and *Arpc5* (5′-GGTGCAGTGTTTCCAAGGCT-3′, forward; 5′-GTACCAGCAACTCCCACTC-3′, reverse; 103-bp amplicon). The resulting products were separated on a 1% agarose gel. Band intensity was quantified using Image Lab (Bio-Rad), and the signal was normalized to *Gapdh* (*5′-*AGGCACCAAGATACTTACAAAAAC*-*3′, forward; 5′- TGTATTGTAACCAGTCATCAGCA-3′, reverse; 193-bp amplicon) as a housekeeping gene.

### CRISPR-Cas9 gene editing of Arp2/3 isoforms

Primary hippocampal neurons were prepared from embryonic day 18 Wistar rat embryos and plated at a density of 50,000 cells per well in 24-well plates containing poly-l-lysine–coated glass coverslips. At DIV3 or DIV10 (depending on the developmental stage of interest), neurons were transfected using Lipofectamine 2000 (Invitrogen), as described for the shRNA experiments. Each transfection was done using 0.5 μg of single guide RNA (sgRNA) plasmid (encoding eGFP) and 1 μg of Cas9-expressing plasmid (with puromycin resistance) per well. sgRNAs were designed using Geneious Prime software on the basis of the Doench 2016 on-target efficiency score, and guides with the highest predicted activity and minimal off-target potential (validated against the rat genome, Rnor_6.0) were selected. The following sgRNA sequences were used: *Arpc1A*: 5′-GACGAAAGCTCACGAGCTGA-3′; *Arpc1B*: 5′-AATGAGAACAAGTTCGCCGT-3′;*Arpc5*: 5′-TTCGTGGACGAGGAGGACGG-3′; *Arpc5L*: 5′-GATGCACAGGGGACATGCTT-3′. For the *Arpc1A* and *Arpc1B* dKO experiments and the corresponding rescue by *Arpc1A* and *Arpc1B*, previously validated sgRNAs were cloned into a single plasmid encoding mCherry and hCas9 (VectorBuilder). Rescue constructs for *Arpc1A* and *Arpc1B* were engineered to be resistant to CRISPR-Cas9–mediated editing and were eGFP-positive. Neurons were cotransfected with CRISPR-Cas9 constructs and the corresponding rescue plasmids at DIV10. Cells were fixed 4 or 5 days posttransfection (corresponding respectively to DIV7 or DIV14). In all experiments, transfections were monitored by eGFP fluorescence; eGFP-positive (transfected) and eGFP-negative (nontransfected) neurons from the same well were analyzed in parallel. In addition, a construct targeting the *Rosa26* locus was used as a negative control (*Rosa*26** locus: 5′-CGCCCATCTTCTAGAAAGAC-3′). In primary neurons, gene disruption efficiency was validated by immunofluorescence using isoform-specific antibodies. Loss of target protein expression in eGFP-positive neurons was quantified by measuring fluorescence intensity in the cell body.

Given that transfection of hippocampal neurons has a low efficiency, to validate CRISPR-Cas9–mediated gene disruption, Rat-2 fibroblasts were transfected with the same sgRNA (0.5 μg) and Cas9-expressing plasmids (1 μg) per well as used for primary neurons, except that the transfection mixture was incubated with the cells for 2 hours before the medium was replaced. Transfections were performed using Lipofectamine 2000 (Invitrogen), following the same protocol described for neurons, except that plasmid DNA and Lipofectamine were diluted in high-glucose Dulbecco’s modified Eagle’s medium. After 48 hours, eGFP-positive cells were sorted by fluorescence-activated cell sorting (BD FACSAria Fusion) and grown in puromycin-containing medium. Genomic DNA was extracted 14 days later using a rapid alkaline lysis method (25 mM NaOH, 98°C, 3 min; neutralized with HCl at room temperature) and used directly for PCR and Sanger sequencing. The following primer pairs were used: *Arpc1A*: 5′-TGCCTTCCTCAGAGATCGCA-3′, forward; 5′-GGCACTCGGAATGTGAGGTG-3′, reverse; *Arpc1B*: 5′-CCCGAGAGTAACCGCATTGT-3′, forward; 5′-TCCGTGTAGTGTCCCCAGAA-3′, reverse; *Arpc5*: 5′- GAAGGTGGACGTGGACGAAT-3′, forward; 5′-GACAGCGCACCTGAGGAATC-3′, reverse; *Arpc5L*: 5′-CAGGTTGGCAGAGGTGTCAT-3′, forward; 5′- CAAGTAGCAGCACACTCCCA-3′, reverse. Clones were analyzed for indels using the ICE analysis tool (Synthego) to confirm frameshift mutations. In both CRISPR-Cas9 KO and shRNA-mediated knockdown experiments, the expression levels of target proteins were quantified using fluorescence intensity measurements in Fiji/ImageJ. For cell body measurements, regions of interest were manually drawn around the soma of transfected (eGFP-positive) and nontransfected (eGFP-negative) neurons, and the mean fluorescence intensity of each target protein was measured. For all conditions, *n* = 3 independent experiments were performed with 15 to 25 neurons per experiment.

### Analysis of AIS length

Hippocampal neurons were transfected at DIV3 with either *Arpc1A* or *Arpc5* shRNAs or with *Arpc1B* or *Arpc5L* sgRNAs and a Cas9-expressing plasmid. At DIV7, neurons were fixed with 2% PFA in PBS for 10 min, incubated with 100% cold methanol on ice for 5 min, quenched with 0.2 M ammonium chloride for 5 min, and blocked for 30 min with 5% normal donkey serum containing 0.05% saponin in PBS. For AIS length measurements, primary antibodies against βIII-tubulin (monoclonal mouse, Promega, G7121, 1:2000) and AnkG (polyclonal rabbit, Synaptic Systems, 386 003, 1:500) were diluted in PBS containing 5% normal donkey serum. Images were acquired at 63× magnification using an AxioImager Z1 microscope (Carl Zeiss, Germany) equipped with an Axiocam MR3.0 camera and Axiovision 4.9 software. The AIS length was measured using the Segmented Line tool in Fiji (ImageJ) by tracing the entire AnkG-positive region from the proximal end to the distal end of the AIS of transfected neurons (eGFP-positive). For every condition, *n =* 3 independent experiments were performed, each with 30 neurons.

### Epifluorescence and STED microscopy

For immunofluorescence of target proteins, images were acquired using a Leica DMI6000 time-lapse epifluorescence microscope (Leica Microsystems) equipped with a 20×/0.40 HCX PL FLUOTAR L CORR Ph1. STED imaging was performed on a Leica Stellaris 8 STED FALCON laser scanning confocal system controlled by LAS X software and with Tau-STED technology (Leica Microsystems). The system was equipped with a pulse white light laser with an 80-MHz repetition rate, a STED depletion pulsed laser line of 775 nm, and two Hybrid Detector X (HyD X, Leica Microsystems) detectors for lifetime-based photon detection. Hippocampal neurons were imaged at a fixed distance of 80 to 100 μm from the cell body using a 93×/1.30–NA (numerical aperture) HC PL APO Glycerol STED WHITE objective (Leica Microsystems) in both confocal and STED modes. Two-dimensional vortex STED images with lateral resolution enhancement were acquired at a pixel size of 20 nm in *xy*, at dwell times of typically 600 ns, and with pinhole set to 0.93 Airy units. The following acquisition settings were applied: 16 × line averaging and detector gating on a Hybrid Detector (HyD, Leica Microsystems) of 0.3 to 6 ns. Tau-STED acquisition was performed with lifetime-based photon separation to suppress background fluorescence and improve contrast. The first STED channel (Abberior STAR 635P) was recorded with 633-nm excitation. The detection bandpass was set to 650 to 750 nm. The second STED channel (Abberior STAR580) was recorded in line sequential mode with 561-nm excitation and a detection window from 580 to 620 nm. All other settings remained constant. We alternatively used an Abberior Instruments “Expert Line” gated STED coupled to a Nikon Ti microscope with an oil-immersion 60× 1.4-NA Plan-Apo objective (Nikon, Lambda Series) and a pinhole size set at 0.8 Airy units. The system features 40-MHz modulated excitation (405, 488, 560, and 640 nm) and depletion (775 nm) lasers and two avalanche photodiode detectors, which were used to gate the detection between ∼700 ps and 8 ns. To quantify MPS periodicity, 1-μm-long lines were drawn along axons in Fiji/ImageJ, and the fluorescence intensity profiles were extracted. For each axon, five lines were analyzed and averaged to obtain a single value per axon. To assess MPS organization, 20- to 23-μm segments in each axon were analyzed. In these segments, the axonal length covered by periodic actin rings relative to the total segment length was calculated and defined as the percentage of MPS abundance. For each condition, *n =* 3 independent experiments were performed, and in each, 6 to 12 axons were analyzed.

### Cross-correlation analysis of actin periodicity

To evaluate the periodic organization of actin rings in axons, cross-correlation analysis was performed on fluorescence intensity profiles extracted from the two-color channels (ARPC2 and βII-spectrin, ARPC2 and phalloidin, or cortactin and ARPC2). One-micrometer-long lines were drawn along axons in Fiji/ImageJ, and the cross-correlation curves were generated. For each axon, five lines were analyzed and averaged to obtain a single curve per axon. A custom macro developed in Fiji/ImageJ was designed to calculate the autocorrelation and normalized cross-correlation functions for each intensity profile (publicly available at https://doi.org/10.5281/zenodo.16878341). The resulting correlation curve identified repeating spatial patterns, where the first side peak (beyond zero lag) indicated both the presence and spacing of periodic actin rings. Axons were considered to show MPS periodicity if the correlation peak exceeded a defined threshold (e.g., *r* > 0.3) and appeared at a spacing of 175 to 205 nm.

### Dual-color single-molecule imaging

For dual-color dSTORM/DNA-PAINT imaging, DIV8 hippocampal neurons were fixed for 30 min using PHEM buffer (60 mM Pipes, 25 mM Hepes, 5 mM EGTA, and 1 mM MgCl_2_, pH 6.9) containing 4% PFA and 0.05% glutaraldehyde. Excess aldehyde was quenched with 50 mM ammonium chloride in PBS for 20 min, and cells were subsequently permeabilized with 0.1% Triton X-100 in PBS for 10 min. After several washes with PBS, cells were blocked with 5% FBS in PBS for 1 hour at 37°C and subjected to immunofluorescence. Samples were incubated with primary antibodies, including polyclonal rabbit anti-ARPC2 (Sigma-Aldrich, HPA008352, 1:50) and monoclonal mouse anti–Ankyrin-G (Santa Cruz Biotechnology, sc-12719, 1:200) in a blocking solution overnight at 4°C. After washing with PBS, cells were incubated with F2-conjugated anti-rabbit IgG nanobodies (Massive Photonics, 3214; batch ID: 3012001) for 1 hour at 37°C. Subsequently, cells were washed and stained with polyclonal donkey anti-rabbit Alexa Fluor 488 secondary antibody (Jackson ImmunoResearch, 711-547-003, 1:500). Afterward, stained samples were washed with PBS containing 5% FBS, rinsed twice with PBS, and postfixed with 4% PFA in PBS for 10 min. Excess aldehyde was quenched with 50 mM ammonium chloride in PBS for 20 min, including a 1:100 dilution of 100-nm gold nanourchins (Cyto Diagnostics, GU-100-20) as fiducial markers. Shortly before imaging, coverslips were incubated with phalloidin-Alexa Fluor Plus 647 for 20 min (Thermo Fisher Scientific, A30107, 0.3 μM), washed thrice, and mounted on Attofluor imaging chambers (Invitrogen, A7816). Single-molecule imaging was performed on a Vutara VXL (Bruker) single-molecule system with biplane 3D imaging modality. The system was operated at 26°C. dSTORM imaging was conducted in imaging buffer containing 100 mM Tris (pH 8.0), 100 mM cysteamine hydrochloride, and protocatechuic acid/protocatechuate-3,4-dioxygenase as an oxygen scavenger system. Forty thousand to 50,000 frames were recorded at a 30-ms exposure time. For DNA-PAINT imaging, 20,000 to 30,000 frames at a 50-ms exposure time were acquired using 1 nM F2-ATTO655 (Massive Photonics) in hybridization buffer (PBS + 500 mM NaCl + 1 mM EDTA + 0.05% Tween 20). Data were analyzed using Bruker SRX (software version 7.0.16). Fiducial markers were used for drift correction and channel alignment.

### Preparation of microelectrode-microfluidic devices and electrophysiology recordings

Microfluidic devices were fabricated by replica molding. Polydimethylsiloxane (PDMS) was prepared using a 10:1 ratio with its curing agent (Sylgard 184 Silicone Elastomer Kit, Dow Corning) and degassed in a vacuum desiccator. Polymerization was carried out at 70°C for 3 hours, after which the PDMS devices were unmolded and trimmed. Medium reservoirs were manually punched using a biopsy punch (Ø 6 mm). The devices consisted of two separate compartments interconnected by 16 microchannels (1000-μm length by 5-μm height by 10-μm width), spaced 200 μm apart. Custom microelectrode array (MEA)-microfluidic chips were prepared as previously described (*9*, *55*). Briefly, microfluidic devices were carefully aligned on planar MEA chips (MultiChannel Systems MCS GmbH, Germany) containing 252 recording electrodes (30 μm in diameter; 200 μm in spacing) arranged in a 16 × 16 grid. Each microchannel encompassed five electrodes to selectively probe propagating axonal signals. Following alignment, chips were air dried in a laminar flow hood and sterilized by ultraviolet light exposure. Then, chips were air plasma cleaned for 1 min at 15 W and sequentially coated with poly-d-lysine (20 μg/ml ml) and laminin (5 μg/ml). One hundred thousand primary hippocampal neurons suspended in 4 μl were seeded in a single compartment. Treatments with drugs were done as detailed above; CK666 and CK689 were used chronically between DIV2 and DIV5, while CK869 was added at DIV5 1 hour before recording. Recording sessions started 1 hour after drug treatment (half-medium change), including a 10-min equilibration period under recording conditions. Spontaneous electrical activity was recorded for 10 min at a sampling rate of 20 kHz using a MEA2100-256 system (MultiChannel Systems MCS, Germany) under controlled temperature (37°C) and a humidified 5% CO_2_ atmosphere (stage-top incubator, ibidi, Germany). Raw signals were high-pass filtered at 200 Hz, and action potentials were detected using a threshold set at 5× the standard deviation (SD) of the electrode noise. Microchannels were classified as active if at least one electrode exhibited an MFR > 0.1 Hz. For each chip, the MFR was calculated by averaging the firing rate across all active microchannels. *n* = 3 to 6 independent μEFs were analyzed per condition.

### In vitro axon degeneration analysis

To quantify the degeneration associated with the loss of ARPC5 and ARPC5L isocomplexes, DIV10 hippocampal neurons were transfected with CRISPR constructs targeting *Arpc5*, *Arpc5L*, or a control plasmid targeting the *Rosa26* locus and maintained in culture until DIV28. Upon fixation, neurons were immunostained for pMLC to label the AIS and identify the axon, and eGFP fluorescence was used to identify transfected neurons. To analyze axon degeneration and calculate the degeneration index, a mask was manually generated for the axon of each eGFP-positive neuron using the Analyze Particles tool in ImageJ (fragment size threshold: 20 to 10,000 px; circularity: 0.2 to 1.0). The ratio of fragmented axonal area to total axonal area was obtained with values ranging from 0 (no degeneration) to 1 (complete fragmentation). *n =* 3 independent experiments and 10 axons per condition were analyzed.

### Axonal transport of mitochondria

To analyze the axonal transport of mitochondria, DIV21 hippocampal neurons (control and *Arpc5L*-depleted) were incubated with medium containing 25 nM MitoTracker Orange CMTMRos (Invitrogen, M7510) for 30 min at 37°C before live imaging. Axons were imaged in a confocal Leica SP8 laser scanning confocal microscope (Leica Microsystems) with LAS X software (version 3.5.7.23225) and an HC FLUOTAR L 25×/0.95 W objective. Excitation was carried out with the 488-nm laser line (to detect eGFP-positive transfected neurons) and the 561-nm laser line (to detect mitochondria). Image acquisition was performed for 3 min with 2- to 2.5-s frame intervals with a pixel size of 0.116 μm. To analyze the axonal transport of moving particles, the MTrackJ plug-in (*56*) from Fiji/ImageJ was used, as described previously (*57*). The percentage of motile mitochondria was obtained by tracking both motile and stationary mitochondria for 3 min. Mitochondria moving in 10 consecutive frames were used to calculate velocities. *n =* 3 independent experiments were performed, and in each, 10 axons were analyzed.

### Analysis of brain slices

Golgi staining was performed in P30 brains from WT (NEX^cre−^; *Arpc5L*^fl/+^) and *Arpc5L* KO (NEX^cre+^; *Arpc5L*^fl/fl^) mice using the FD Rapid GolgiStain Kit (FD Neurotechnologies; PK401A), according to the manufacturer’s instructions. Briefly, mouse brains were immersed in the impregnation solution (solutions A and B), stored for 2 weeks at room temperature in the dark, then transferred to solution C, and kept at 4°C in the dark for one extra week. Subsequently, brains were impregnated in 3% low-melting agarose (NZYTech, MB12301) and sectioned using a vibratome (Leica VT 1200S) at a thickness of 200 μm, and slices were stained with solutions D and E. Tissue clearing was performed using the Ubiclear protocol (Idylle, IDY-UBI-L), slices were mounted in a postclearing solution, and images were acquired with a MIchrome 5 Pro camera (Tucsen) mounted on an upright bright-field microscope (Olympus CX31). Given the dense and highly complex network of processes in the hippocampus that precluded reliable morphological quantification, the cortex was examined. For each genotype, *n* = 5 animals were analyzed. For each brain, 12 images were acquired from the cortex at 40× magnification. The number of neuronal processes exhibiting swellings was quantified in each image, and results were expressed as density per μm^2^.

### Statistical analyses

Sample sizes were estimated on the basis of expected effect size, power, and significance level and according to the previous experience of our group. Researchers were blinded to experimental conditions and animal genotypes. All quantitative data are presented as the means ± standard error of the mean (SEM), unless otherwise stated. Statistical analyses were performed using GraphPad Prism. For comparisons between two groups, unpaired two-tailed Student’s *t* test was used. For experiments involving more than two conditions or time points, a one-way analysis of variance (ANOVA) was performed followed by Tukey’s post hoc test for multiple comparisons, as appropriate. In all cases, statistical significance was set at *P* < 0.05. Exact *P* values and sample sizes are indicated in the figure legends.
