# Supplementary material for: Distinct Arp2/3 isocomplexes drive axonal actin ring assembly and integrity
Source: Sci Adv. 2026 Sep 25;12(39):eaec9522. doi: 10.1126/sciadv.aec9522 (PMC13614374; doi:10.1126/sciadv.aec9522)
Supplement: Supplementary file 1 [file sciadv.aec9522_sm.pdf]

Science Advances
AAAS

Supplementary Materials for

Distinct Arp2/3 isocomplexes drive axonal actin ring assembly and integrity

Ana Rita Costa *et al.*

Corresponding author: Ana Rita Costa, arcosta@i3s.up.pt; Monica M. Sousa, msousa@i3s.up.pt

Sci. Adv. **12**, eaec9522 (2026)
DOI: 10.1126/sciadv.aec9522

The PDF file includes:

Figs. S1 to S5
Legend for data file S1

Other Supplementary Material for this manuscript includes the following:

Data file S1

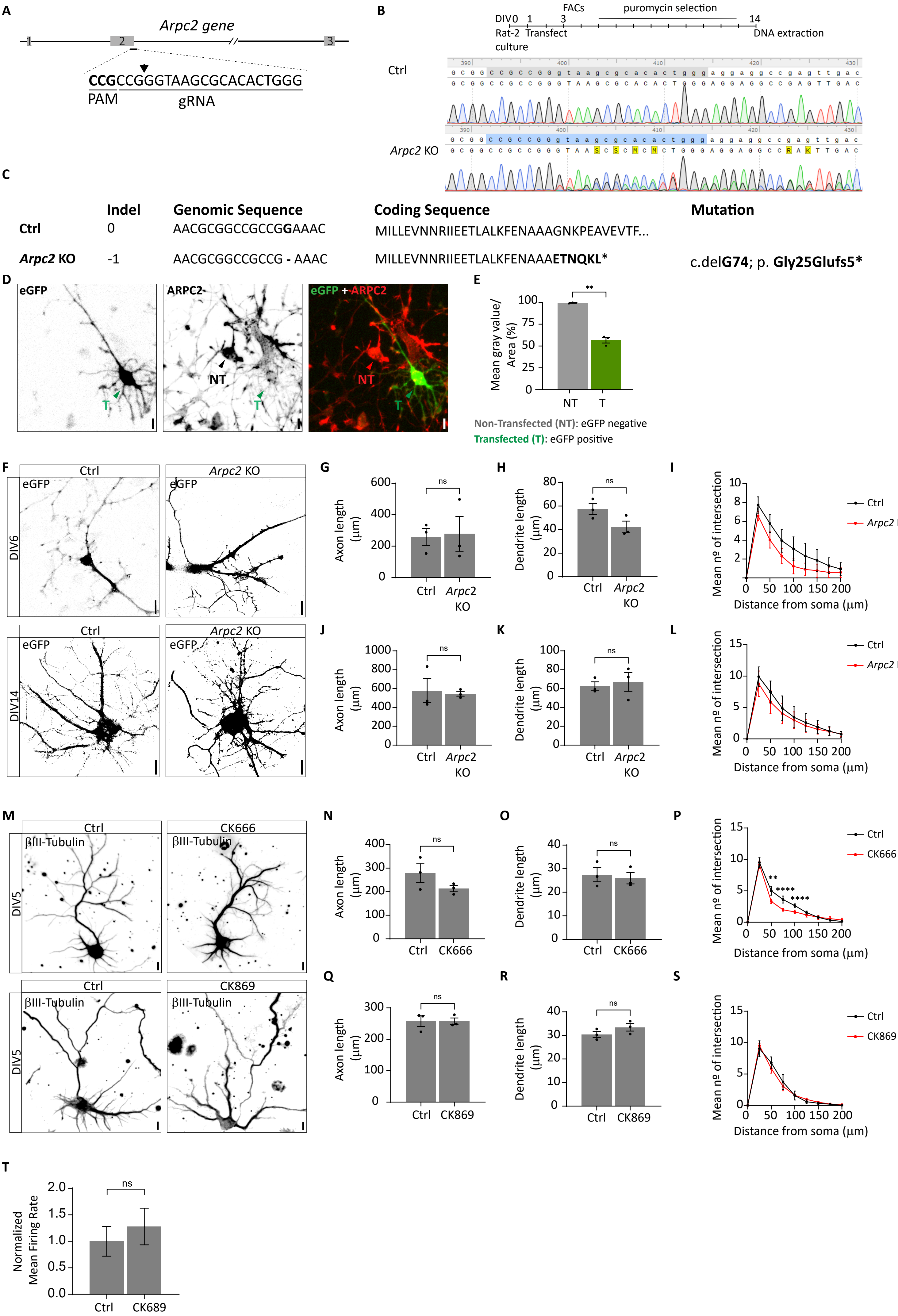

A
Arpc2 gene
1
2
3
CCGCCGGGTAAGCGCACACTGGG
PAM
gRNA
B
FACs
puromycin selection
DIV0 1 3 14
Rat-2 culture
Transfect
DNA extraction
Ctrl
Arpc2 KO
C
Indel
Genomic Sequence
Coding Sequence
Mutation
Ctrl
0
AACGCGGCCGCCGGAAAC
MILLEVNNRIIEETLALKFENAAAGNKPEAVEVTF...
Arpc2 KO
-1
AACGCGGCCGCCG - AAAC
MILLEVNNRIIEETLALKFENAAAETNQKL*
c.delG74; p. Gly25Glufs5*
D
eGFP
ARPC2
eGFP + ARPC2
NT
T
E
Mean gray value/ Area (%)
NT
T
Non-Transfected (NT): eGFP negative
Transfected (T): eGFP positive
F
Ctrl
Arpc2 KO
DIV6
DIV14
eGFP
G
Axon length (µm)
H
Dendrite length (µm)
I
Mean nº of intersection
Distance from soma (µm)
Ctrl
Arpc2 KO
J
K
L
M
βIII-Tubulin
CK666
CK869
DIV5
N
O
P
Q
R
S
T
Normalized Mean Firing Rate
Ctrl
CK689
ns

Supplementary Text

Fig. S1. Validation of CRISPR/Cas9-mediated KO of *Arpc2*

(A) Schematic representation of the *Arpc2* gene showing the location of the guide RNA (gRNA) sequence and PAM site used for CRISPR targeting. **(B)** Timeline and Sanger sequencing chromatogram showing the wild-type (WT) and *Arpc2* KO sequences following CRISPR/Cas9 editing in Rat-2 cells. **(C)** Alignment of the *Arpc2* genomic sequences in control and *Arpc2* KO clones reveals a one-base-pair deletion (c.delG74) causing a frameshift and premature stop codon (p.Gly25Glufs*5). Predicted amino acid sequences of wild-type and mutant ARPC2, indicating truncation of the protein caused by the introduced indel. **(D)** Immunofluorescence images of DIV6 hippocampal neurons transfected (T) with the CRISPR targeting *Arpc2* (eGFP positive), and non-transfected (NT- eGFP negative) neighbouring neurons. Scale bar: 10 μm. **(E)** Quantification of ARPC2 fluorescence signal intensity in the neuronal cell body; Data represent *n*=3 independent experiments, Ctrl and *Arpc*2 KO, 25 and 20 neurons analyzed per experiment, respectively. **(F)** Representative confocal images of DIV6 and DIV14 hippocampal neurons transfected with a CRISPR targeting *Rosa26* (Ctrl) or *Arpc2* (*Arpc2* KO) at DIV3 and DIV10, respectively. **(G-L)** Quantification of axon length, dendrite length, and branching related to (F), at DIV6 (G-I) and DIV14 (J-L). *n*=3 independent experiments; 20 neurons were quantified per length analysis experiment and 30 per branching analysis experiment. **(M)** Representative βIII-tubulin immunofluorescence images of DIV5 hippocampal neurons treated with DMSO (Ctrl), CK666, or CK869; Scale bar: 10 μm. **(N-S)** Quantification of axon length, dendrite length and branching following CK666 (N-P) and CK869 (Q-S) treatment. *n*=3 independent experiments; 20 neurons were quantified per length analysis experiment and 30 per branching analysis experiment. **(T)** Normalized mean firing rate of DIV5 primary hippocampal neurons treated either with control vehicle (DMSO; Ctrl) or the inactive inhibitor of Arp2/3, CK689 (15 μM); *n*=3 independent μEFs/condition. Data are presented as mean ± s.e.m.; unpaired *t*-test and one-way ANOVA followed by the Tukey test (I and S). **** $P \leq 0.0001$, ** $P \leq 0.01$, * $P \leq 0.05$ and ns- non-significant.

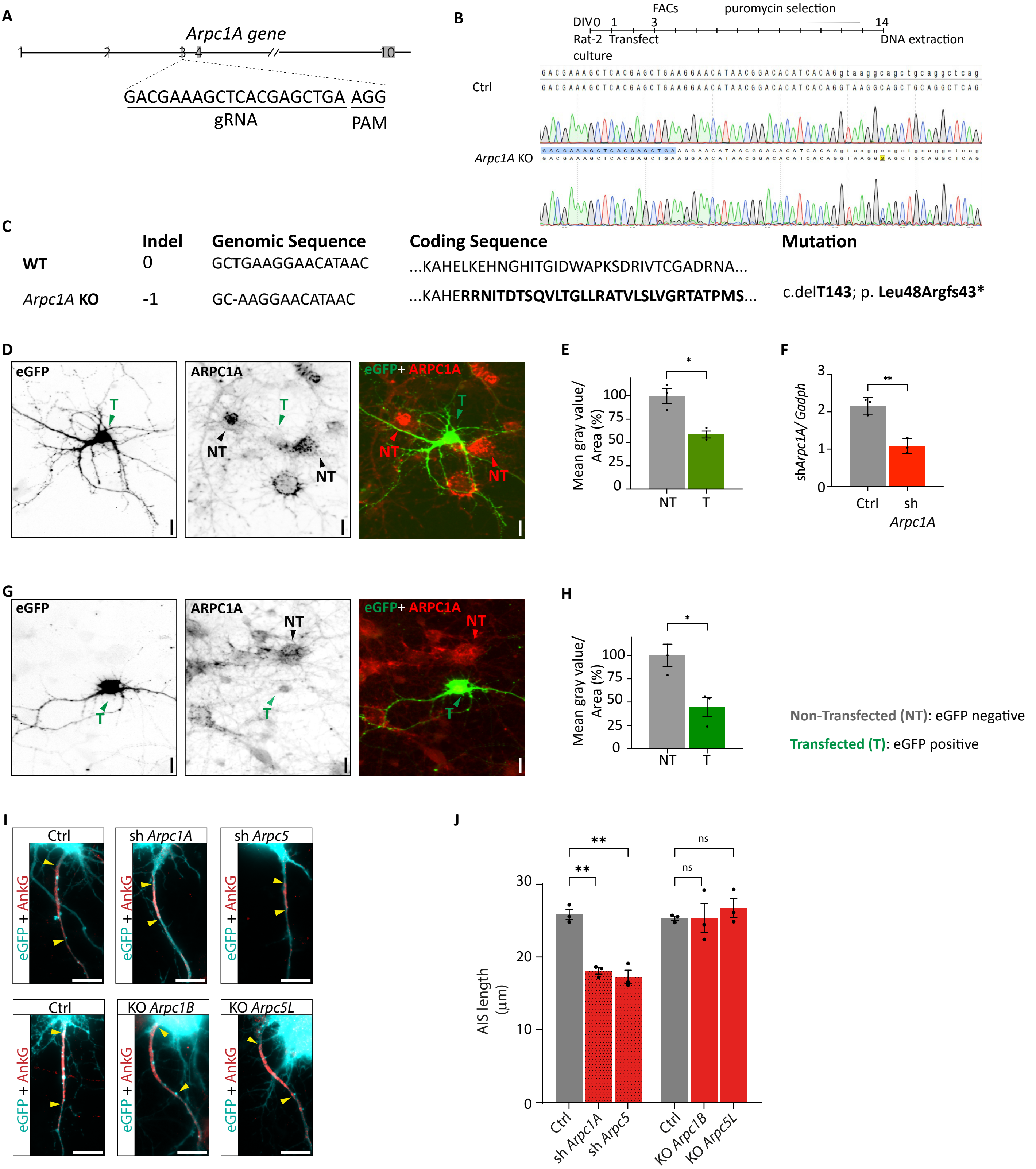
A
Arpc1A gene
1
2
3
4
10
GACGAAAGCTCACGAGCTGA AGG
gRNA
PAM
B
FACs
puromycin selection
DIV0 1 3 14
Rat-2 culture
Transfect
DNA extraction
Ctrl
Arpc1A KO
C
Indel
Genomic Sequence
Coding Sequence
Mutation
WT
0
GCTGAAGGAACATAAC
...KAHELKEHNGHITGIDWAPKSDRIVTCGADRNA...
Arpc1A KO
-1
GC-AAGGAACATAAC
...KAHERRNITDTSQVLTGLLRATVLSLVGRTATPMS...
c.delT143; p. Leu48Argfs43*
D
eGFP
ARPC1A
eGFP+ ARPC1A
T
NT
E
Mean gray value/ Area (%)
NT
T
F
shArpc1A/ Gadph
Ctrl
sh Arpc1A
G
eGFP
ARPC1A
eGFP+ ARPC1A
T
NT
H
Mean gray value/ Area (%)
NT
T
Non-Transfected (NT): eGFP negative
Transfected (T): eGFP positive
I
Ctrl
sh Arpc1A
sh Arpc5
KO Arpc1B
KO Arpc5L
eGFP + AnkG
J
AIS length (µm)
Ctrl
sh Arpc1A
sh Arpc5
Ctrl
KO Arpc1B
KO Arpc5L
ns

Fig. S2. Validation of *Arpc1A* shRNA-mediated downregulation and CRISPR-Cas9-mediated gene editing

(A) Scheme of the *Arpc1A* gene showing the location of the CRISPR guide RNA and PAM site. **(B)** Timeline and Sanger sequencing chromatograms of wild-type (WT) and *Arpc1A* knockout (KO) alleles in Rat-2 cells transfected with CRISPR/Cas9 targeting *Arpc1A*. **(C)** Alignment of the *Arpc1A* genomic sequences showing a single base-pair deletion (–1 bp) in the KO allele, resulting in a frameshift mutation: c.delT143 leading to p.Leu48Argfs*43 and the respective predicted amino acid sequences of WT and mutant ARPC1A proteins, showing the effect of the frameshift. **(D)** Immunofluorescence of DIV7 hippocampal neurons transfected at DIV3 with a shRNA targeting ARPC1A. **Left**: eGFP channel showing transfected neuron (T; eGFP-positive; green arrowheads); **Middle**: ARPC1A immunofluorescence; green arrowhead highlights transfected neuron and red arrowheads non-transfected eGFP-negative neighbouring neurons; **Right**: merged. Scale bar: 10 µm. **(E)** Quantification of ARPC1A fluorescence signal intensity in the neuronal cell body relative to (D) of transfected and non-transfected neurons. *n* = 3 independent experiments, 20–25 neurons analyzed per experiment: Ctrl and sh*Arpc1A,* 25 and 20 neurons analyzed per experiment, respectively. **(F)** qPCR analysis of Rat-2 cells transfected with a scramble (Ctrl) or a shRNA targeting *Arpc1A* (sh *Arpc1A*). *n=3* independent experiments. **(G)** Immunofluorescence of DIV14 hippocampal neurons transfected at DIV10 with the CRISPR targeting *Arpc1A*. **Left**: eGFP channel showing transfected (T) neuron (highlighted with green arrowhead); **Middle**: ARPC1A immunofluorescence; transfected neuron is highlighted by green arrowhead and non-transfected neighbouring neuron by red arrowheads; **Right:** merge. Scale bar: 10 µm. **(H)** Quantification of ARPC1A fluorescence signal intensity per unit area in the neuronal cell body related to (G). *n* = 3 independent experiments, 18–20 neurons analyzed per experiment: Ctrl and *Arpc1A* KO, 20 and 18 neurons analyzed per experiment, respectively. **(I)** Representative AnkG immunofluorescence images of DIV7 hippocampal neurons transfected at DIV3 with either control constructs (scramble shRNA or CRISPR targeting *Rosa26*), shRNAs targeting *Arpc1A* or *Arpc5*, or CRISPR constructs targeting *Arpc1B* or *Arpc5L*. Arrowheads indicate the proximal and distal boundaries of the AIS used for length measurements. Scale bar: 10 µm. **(J)** Quantification of the AIS length related to (I). *n* = 3 independent experiments, *n*=30 neurons analyzed per experiment. Data are presented as mean ± s.e.m.; unpaired *t*-test (E, F and H) and one-way ANOVA followed by the Tukey test (J). ** P ≤ 0.01, * P ≤ 0.05 and ns- non-significant.

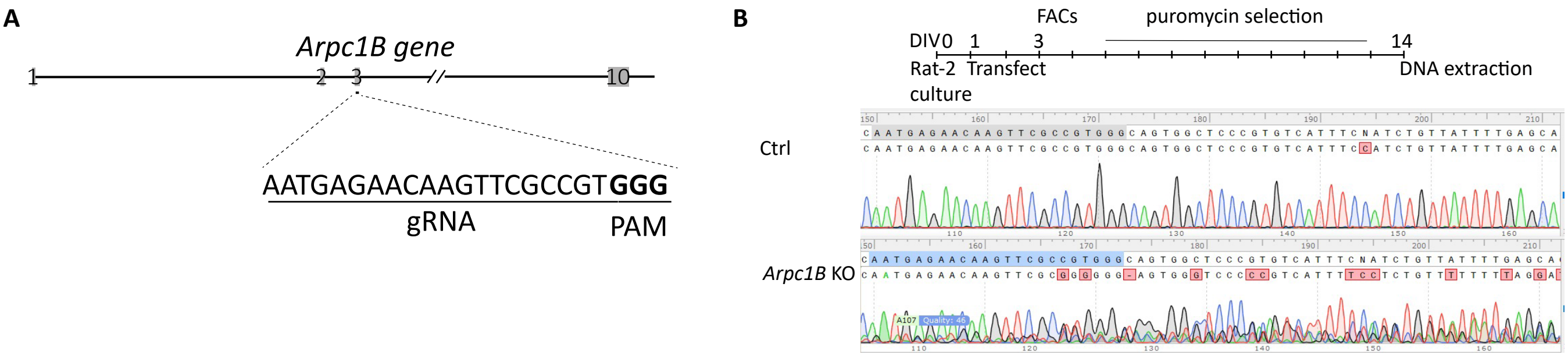

C

	Indel	Genomic Sequence	Coding Sequence	Mutation
**Ctrl**	0	AGAACAAGTTCGCCGT	...NENKFAVGSGSRVISICYFEQEND...	
***Arpc1B* KO**	-1	AGAACAAGTTCGCCG-	...NENKFA**GAVAPVSFPSVILSRRMT...**	c.del**T342**; p. **Val113Glyfs80***

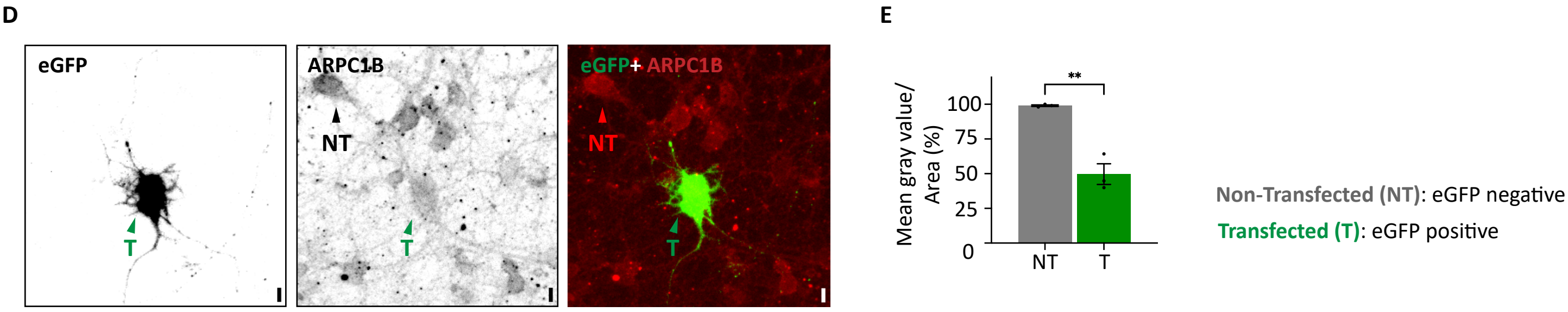

Fig. S3. Validation of *Arpc1B* CRISPR/Cas9 editing in hippocampal neurons **(A)** Schematic representation of the *Arpc1B* gene indicating the location of the guide RNA (gRNA) and PAM sequence used for CRISPR/Cas9 editing. **(B)** Timeline and Sanger sequencing chromatograms of WT and mutant *Arpc1B* genomic sequences showing a one-base-pair deletion in the KO condition (c.delT342), confirmed by Sanger sequencing. **(C)** Alignment of the *Arpc1B* genomic DNA revealing the deletion and resulting frameshift mutation and the predicted amino acid sequence showing that the mutation induces a frameshift leading to a premature stop codon (p.Val113Glyfs*80), truncating the ARPC1B protein. **(D)** Immunofluorescence of DIV14 hippocampal neurons transfected at DIV10 with a CRISPR targeting *Arpc1B*. **Left**: eGFP channel showing transfected (T) neuron (eGFP-positive; green arrowheads). **Middle**: ARPC1B immunofluorescence; T- transfected eGFP-positive neuron, highlighted by green arrowhead and NT, non-transfected eGFP-negative neighbouring neuron, highlighted by red arrowheads. **Right**: merge. Scale bar: 10 µm. (E) Quantification of ARPC1B fluorescence signal intensity in the neuronal cell body, relative to (D). *n*=3 independent experiments; 25 neurons analyzed per experiment. Data are presented as mean ± s.e.m.; unpaired *t*-test. ** $P \leq 0.01$ and ns- non-significant.

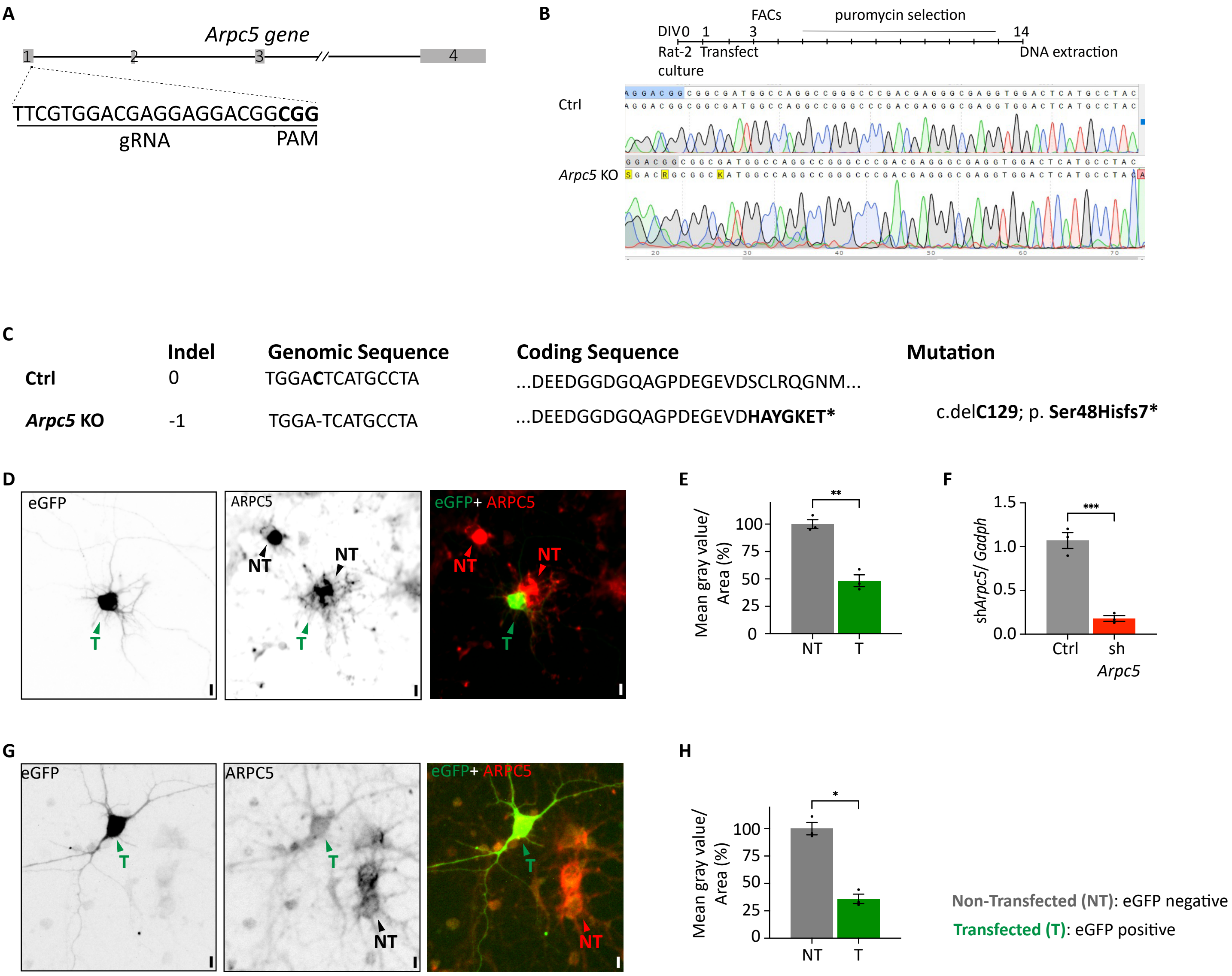
A
Arpc5 gene
1
2
3
4
TTCGTGGACGAGGAGGACGGCGG
gRNA
PAM
B
FACs
puromycin selection
DIV0
1
3
14
Rat-2 culture
Transfect
DNA extraction
Ctrl
Arpc5 KO
C
Indel
Genomic Sequence
Coding Sequence
Mutation
Ctrl
0
TGGACTCATGCCTA
...DEEDGGDGQAGPDEGEVDSCLRQGNM...
Arpc5 KO
-1
TGGA-TCATGCCTA
...DEEDGGDGQAGPDEGEVDHAYGKET*
c.delC129; p. Ser48Hisfs7*
D
eGFP
ARPC5
eGFP+ ARPC5
T
NT
E
Mean gray value/ Area (%)
100
75
50
25
0
**
NT
T
F
shArpc5/ Gadph
1.5
1.0
0.5
0.0
***
Ctrl
sh
Arpc5
G
eGFP
ARPC5
eGFP+ ARPC5
T
NT
H
Mean gray value/ Area (%)
100
75
50
25
0
*
NT
T
Non-Transfected (NT): eGFP negative
Transfected (T): eGFP positive

Fig. S4. Validation of *Arpc5* shRNA-mediated downregulation and CRISPR-Cas9-mediated editing

(A) Schematic representation of the *Arpc5* gene showing the location of the CRISPR guide RNA and PAM site. **(B)** Timeline and Sanger sequencing chromatograms of wild-type (WT) and *Arpc5* KO alleles in Rat-2 cells transfected with *Arpc5* targeting CRISPR/Cas9. **(C)** Alignment of genomic sequences of *Arpc5* showing a single base-pair deletion (–1 bp) in the KO allele, resulting in a frameshift mutation: c.delC129 leading to p.Ser48Hisfs7* and the respective predicted amino acid sequences of WT and mutant ARPC5 proteins, showing the effect of the frameshift. **(D)** Immunofluorescence of DIV7 hippocampal neurons transfected at DIV3 with a shRNA targeting ARPC5. **Left**: eGFP channel showing neuron transfected with the shRNA targeting *Arpc5* (T, eGFP-positive; highlighted with green arrowhead). **Middle**: ARPC5 immunostaining, eGFP-positive transfected neuron highlighted by green arrowhead and neighbouring non-transfected neuron (NT, eGFP-negative), highlighted by red arrowheads. **Right**: merge. Scale bar: 10 µm. **(E)** Quantification of ARPC5 fluorescence signal intensity in the neuronal cell body, relative to (D). n=3 independent experiments; Ctrl and sh*Arpc5,* 22 and 20 neurons analysed per experiment, respectively. **(F)** qRT-PCR analysis of Rat-2 cells transfected with a scramble (Ctrl) or a shRNA targeting *Arpc5* (sh *Arpc*5). **(G)** Immunofluorescence of DIV7 hippocampal neurons where *Arpc5* was edited at DIV3. **Left**: eGFP channel showing neuron transfected (T) with a CRISPR-targeting *Arpc5* (eGFP-positive; green arrowheads) and neighbouring non-transfected neuron (NT; eGFP-negative; red arrowheads). **Middle**: ARPC5 immunofluorescence; transfected (T, eGFP-positive; green arrowheads) and non-transfected (NT, eGFP-negative; red arrowheads) neighbouring neurons. **Right**: merge. Scale bar: 10 µm. **(H)** Quantification of ARPC5 fluorescence signal intensity in the neuronal cell body, relative to (G). n=3 independent experiments; 20 neurons analyzed per experiment. Data are presented as mean ± s.e.m.; unpaired t-test. *** $P \leq 0.001$, ** $P \leq 0.01$, * $P \leq 0.05$ and ns- non-significant.

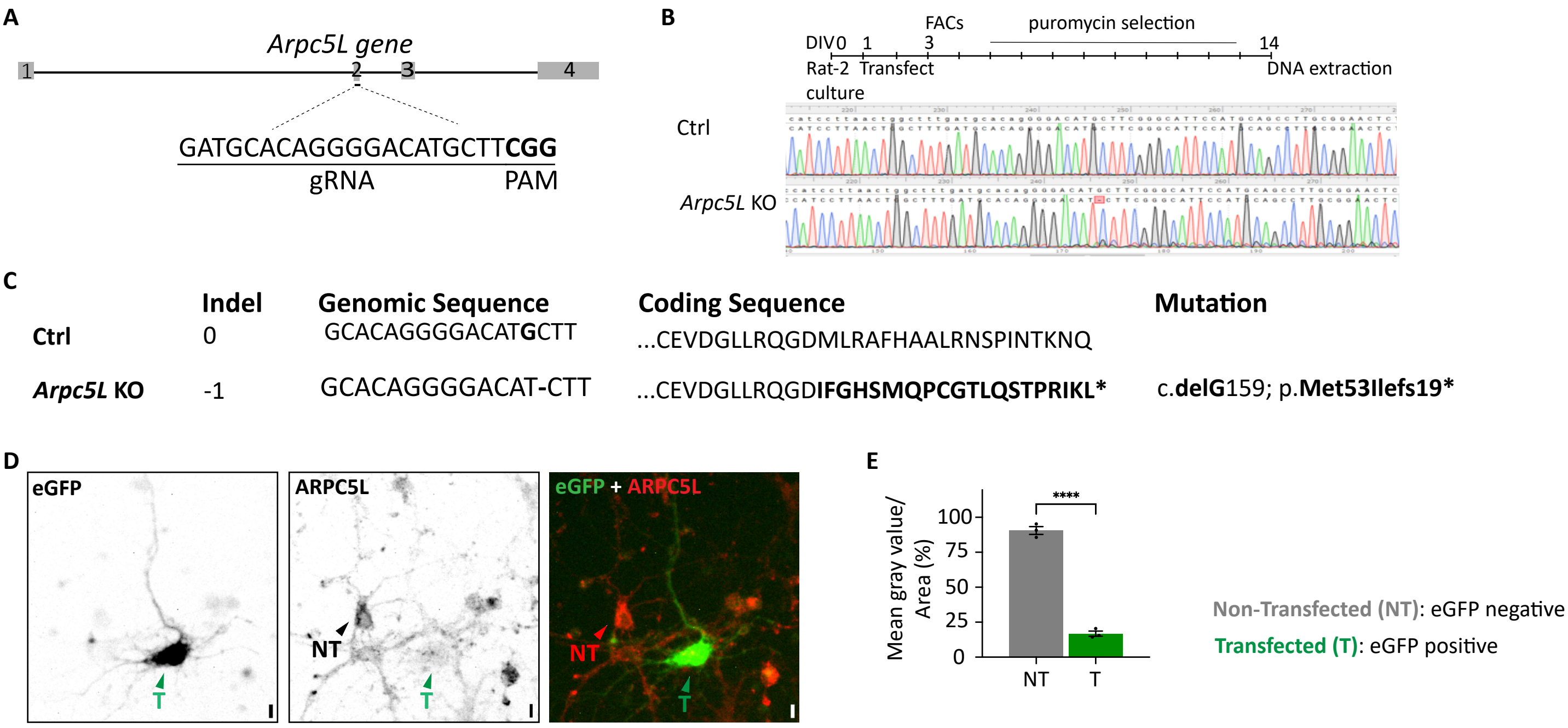
A
Arpc5L gene
1
2
3
4
GATGCACAGGGGACATGCTTCGG
gRNA
PAM
B
DIV0
1
Rat-2 culture
Transfect
FACs
3
puromycin selection
14
DNA extraction
Ctrl
Arpc5L KO
C
Indel
Genomic Sequence
Coding Sequence
Mutation
Ctrl
0
GCACAGGGGACATGCTT
...CEVDGLLRQGDMLRAFHAALRNSPINTKNQ
Arpc5L KO
-1
GCACAGGGGACAT-CTT
...CEVDGLLRQGDIFGHSMQPCGTLQSTPRIKL*
c.delG159; p.Met53Ilefs19*
D
eGFP
ARPC5L
eGFP + ARPC5L
NT
T
E
Mean gray value/ Area (%)
100
75
50
25
0
****
NT
T
Non-Transfected (NT): eGFP negative
Transfected (T): eGFP positive

Fig. S5. Validation of *Arpc5L* editing through CRISPR/Cas9 KO

(A) Schematic representation of the *Arpc5L* gene indicating the location of the guide RNA (gRNA) and PAM sequence used for CRISPR/Cas9 editing. **(B)** Experimental timeline of CRISPR/Cas9 targeting in Rat-2 cells. Wild-type (WT) and mutant *Arpc5L* genomic sequences showing a one-base-pair deletion in the KO condition (c.159delG), confirmed by Sanger sequencing. **(C)** Genomic alignment showing the −1 bp indel in the *Arpc5L* KO allele relative to WT and the predicted protein sequence from the KO allele reveals a frameshift mutation resulting in a premature stop codon (p.Met53Ilefs*19), truncating the ARPC5L protein. **(D)** Immunofluorescence of DIV14 hippocampal neurons transfected at DIV10 with a CRISPR targeting *Arpc5L*. **Left**: eGFP channel showing neuron transfected (T) with a CRISPR targeting *Arpc5L* (eGFP-positive; green arrowheads). **Middle**: ARPC5L immunofluorescence; transfected (T, eGFP-positive, green arrowheads) and non-transfected (NT, eGFP-negative; red arrowheads) neighbouring neurons are highlighted. **Right**: merge. Scale bar: 10 µm. **(E)** Quantification of ARPC5L fluorescence signal intensity in the neuronal cell body, relative to (D). *n* =3 independent experiments; Ctrl (*Rosa*) and *Arpc5L* KO, 20 and 15 neurons analysed per experiment, respectively. Data are presented as mean ± s.e.m.; unpaired *t*-test. **** P ≤ 0.0001.

Data file S1. Source data for the quantitative analyses presented in the main figures Excel file containing the individual biological replicate values, experimental conditions, and number of independent experiments underlying the quantitative analyses presented in the main figures.
